# Growth factor independence underpins a paroxysmal, aggressive Wnt5a^High^/EphA2^Low^ phenotype in glioblastoma stem cells, conducive to experimental combinatorial therapy

**DOI:** 10.1186/s13046-022-02333-1

**Published:** 2022-04-12

**Authors:** Nadia Trivieri, Alberto Visioli, Gandino Mencarelli, Maria Grazia Cariglia, Laura Marongiu, Riccardo Pracella, Fabrizio Giani, Amata Amy Soriano, Chiara Barile, Laura Cajola, Massimiliano Copetti, Orazio Palumbo, Federico Legnani, Francesco DiMeco, Leonardo Gorgoglione, Angelo L. Vescovi, Elena Binda

**Affiliations:** 1 Cancer Stem Cells Unit, Institute for Stem Cell Biology, Regenerative Medicine and Innovative Therapeutics (ISBReMIT), IRCSS Casa Sollievo della Sofferenza, Opera di San Pio da Pietrelcina, 71013 San Giovanni Rotondo, FG Italy; 2 StemGen SpA, Milan, Italy; 3 Department of Biotechnology and Biosciences, University of Milano-Bicocca, Milan, Italy; 4 Biostatistics Unit, IRCCS Casa Sollievo della Sofferenza, San Giovanni Rotondo, Italy; 5 Medical Genetics Unit, IRCCS Casa Sollievo della Sofferenza, San Giovanni Rotondo, Italy; 6 Department of Neurosurgery, National Neurologic Institute IRCCS C. Besta, Milan, Italy; 7 Department of Neurosurgery, John Hopkins University, Baltimore, MD USA; 8 Neurosurgery Unit, IRCCS Casa Sollievo della Sofferenza, San Giovanni Rotondo, Italy; 9 Scientific Directorate, IRCCS Casa Sollievo della Sofferenza, FG San Giovanni Rotondo, Italy; 10 Hyperstem SA, Lugano, Switzerland

**Keywords:** Glioblastoma, Mitogen-independence, GBM cancer stem cells (GSCs), GSCs biology and biomarkers, Anti-GSCs patient-tailored strategies

## Abstract

**Background:**

Glioblastoma multiforme (GBM) is an incurable tumor, with a median survival rate of only 14–15 months. Along with heterogeneity and unregulated growth, a central matter in dealing with GBMs is cell invasiveness. Thus, improving prognosis requires finding new agents to inhibit key multiple pathways, even simultaneously. A subset of GBM stem-like cells (GSCs) may account for tumorigenicity, representing, through their pathways, the proper cellular target in the therapeutics of glioblastomas. GSCs cells are routinely enriched and expanded due to continuous exposure to specific growth factors, which might alter some of their intrinsic characteristic and hide therapeutically relevant traits.

**Methods:**

By removing exogenous growth factors stimulation, here we isolated and characterized a subset of GSCs with a “mitogen-independent” phenotype (I-GSCs) from patient’s tumor specimens. Differential side-by-side comparative functional and molecular analyses were performed either in vitro or in vivo on these cells versus their classical growth factor (GF)-dependent counterpart (D-GSCs) as well as their tissue of origin. This was performed to pinpoint the inherent GSCs’ critical regulators, with particular emphasis on those involved in spreading and tumorigenic potential. Transcriptomic fingerprints were pointed out by ANOVA with Benjamini-Hochberg False Discovery Rate (FDR) and association of copy number alterations or somatic mutations was determined by comparing each subgroup with a two-tailed Fisher’s exact test. The combined effects of interacting in vitro and in vivo with two emerging GSCs’ key regulators, such as Wnt5a and EphA2, were then predicted under in vivo experimental settings that are conducive to clinical applications. In vivo comparisons were carried out in mouse-human xenografts GBM model by a hierarchical linear model for repeated measurements and Dunnett’s multiple comparison test with the distribution of survival compared by Kaplan–Meier method.

**Results:**

Here, we assessed that a subset of GSCs from high-grade gliomas is self-sufficient in the activation of regulatory growth signaling. Furthermore, while constitutively present within the same GBM tissue, these GF-independent GSCs cells were endowed with a distinctive functional and molecular repertoire, defined by highly aggressive Wnt5a^High^/EphA2^Low^ profile, as opposed to Wnt5a^Low^/EphA2^High^ expression in sibling D-GSCs. Regardless of their GBM subtype of origin, I-GSCs, are endowed with a raised in vivo tumorigenic potential than matched D-GSCs, which were fast-growing ex-vivo but less lethal and invasive in vivo. Also, the malignant I-GSCs’ transcriptomic fingerprint faithfully mirrored the original tumor, bringing into evidence key regulators of invasiveness, angiogenesis and immuno-modulators, which became candidates for glioma diagnostic/prognostic markers and therapeutic targets. Particularly, simultaneously counteracting the activity of the tissue invasive mediator Wnt5a and EphA2 tyrosine kinase receptor addictively hindered GSCs’ tumorigenic and invasive ability, thus increasing survival.

**Conclusion:**

We show how the preservation of a mitogen-independent phenotype in GSCs plays a central role in determining the exacerbated tumorigenic and high mobility features distinctive of GBM. The exploitation of the I-GSCs' peculiar features shown here offers new ways to identify novel, GSCs-specific effectors, whose modulation can be used in order to identify novel, potential molecular therapeutic targets. Furthermore, we show how the combined use of PepA, the anti-Wnt5a drug, and of ephrinA1-Fc to can hinder GSCs’ lethality in a clinically relevant xenogeneic in vivo model thus being conducive to perspective, novel combinatorial clinical application.

**Supplementary Information:**

The online version contains supplementary material available at 10.1186/s13046-022-02333-1.

## Background

IDH1 wild-type glioblastoma (GBM) is the most common and malignant among gliomas [1]. Even upon aggressive surgery, radiation- and chemotherapy, this tumor inevitably recurs and dismal overall survival of GBM patients persists [2, 3]. A critical factor in this situation is the extensive cellular heterogeneity of this cancer, both intratumoral and interpatient [4–6]. This scenario led to the development of single-agent molecularly targeted therapies, to provide treatments that are more effective and less toxic than conventional chemotherapy [7–9]. Notwithstanding, recent findings pointed to the existence in GBM cells of multiple, redundant or converging signaling pathways, that critically underpin their striking tumorigenic and invasive capacity [10, 11]. This notion is now lending to multipronged approaches in which the combined or simultaneous use of multiple agents is viewed as crucial for efficacious anti-GBM therapies [12, 13]. Such a scenario is compounded by the discovery that only a relative small subset of idiosyncratic cells in GBMs does possess actual tumor-initiating and propagating ability and resistance to standard, multimodal treatments, thereby determining recurrence after therapy, even at the clonal level [14–18]. These GBM stem-like cells (GSCs) now represent a golden cellular target in glioblastoma treatment [19–21].

Multiple studies reported the existence of three or four different transcriptionally defined molecular GBM subtypes, which underlie the GBM malignant cellular heterogeneity, according to their genetic, genomic and functional characteristics [4, 22–26] namely Proneural, Neural, Classical or Proliferative and Mesenchymal. The Proneural subclass is defined by genes implicated in neurogenesis and oligodendrocytic development genes and harbors frequent PDGFRA amplification and point mutations in IDH1 and TP53. The Neural subtype is characterized by the expression of neuron markers. In contrast, Classical or Proliferative subclass is defined by genes associated with a high rate of proliferation, frequent EGFR amplification and EGFRvIII mutations and CDKN2A deletion, whereas the Mesenchymal subgroup by extracellular matrix/invasion-related genes and mesenchymal markers, deletion of NF1, TP53, and PTEN genes and increased necrosis, angiogenesis and inflammation, respectively. It has been also described the existence of four distinct cellular programs among GBM cells [25] and that GSCs display different level of stem cell markers in correlation with their GBM subcluster of origin [27]. All of these stratifications underpin functional inter-cluster variances in GBM and pinpoint that GSCs remain poorly understood and elusive biological entities and therapeutic targets.

A key notion is now consolidating that GBM might recapitulate a normal neurodevelopmental hierarchy and several neural niche effectors or developmental master factors, including the tyrosine kinase receptor EphA2, are implicated in the regulation of GSCs self-renewal and tumor-propagating potential as well [28–32]. The concept of neurogenic effectors regulating GSCs was recently reinforced by the identification of *WNT5A* gene as a master switch that controls the differentiation/proliferation balance of these cells and, thus, their lethality and intracranial invasion capacity [27, 33]. Hence, therapeutic approaches targeting these selective signaling pathways might effectively deplete the GSCs population within GBMs.

Importantly, GSCs share some key functional characteristics and regulatory cues with normal neural stem cells (NSCs) [34–36]. Thus, the same families of growth factors, cytokines and chemokines that have been described to play a fundamental role in controlling the proliferation and fate of NSCs, have been found to modulate GSCs activity in GBMs [37, 38]. Unsurprisingly, the deregulation of these signaling pathways activated by GFs happens to be a critical element in the acquisition of tumorigenic features by transformed GSCs [39], not only in GBMs [40] but also in hematopoietic malignancies [41]. Here we focus on a peculiar aspect of this phenomenon in GSCs. Unlike normal NSCs, which are inherently dependent on exogenous GFs and undergo cell cycle withdrawal and differentiation upon GFs starvation [42, 43], gliomatous transformation may bestow the ability to sustain proliferation in the absence of mitogenic stimulation upon some GBM cells, also as triggered by exogenous factors [39, 44, 45]. This was confirmed by the isolation and cloning of GBM GSCs in the absence of exogenous GFs [46] and by the initial evidence of a direct association between GF-independence and GBM subtypes, although in short term cultures [26]. The acquisition of a seemingly “GF-independent” phenotype might be underpinned by specific alterations in the pattern of gene expression in GSCs [47].

The classical, standard procedure for isolation and propagation of GSCs entails the use of saturating concentrations of exogenous EGF and FGF2 [14, 48, 49]. The identification of a pool of prospective GSCs which appears to be independent from these GFs raises numerous and enticing, yet unanswered questions, as to their presence in the various GBM subtypes, their phenotype and their functional, molecular and pathophysiological characteristics.

Here, we report that GSCs with unlimited self-renewal capacity, that are inherently independent from exogenous growth factors, can be isolated from all the GBM subtypes. These GSCs possess functional properties that are strikingly different from classical GSCs at the level of their molecular, functional and tumorigenic potential. We show that the exacerbated tumor-propagating capacity and invasive potential of these GFs-independent GSCs is associated to a distinctive transcriptional program that is strictly reminiscent of their tissue of origin. GF-independent GSCs are defined by a specific Wnt5a^High^/EphA2^Low^ immunophenotype.

Simultaneously counteracting Wnt5a and EphA2 activity in orthotopic settings in vivo defines an effective combinatorial putative experimental therapeutic approach that efficaciously antagonizes growth, spread and lethality of GSCs cells.

## Methods

*Immunochemistry, Reagents, Flow cytometry, Cell Cycle Analysis, Exploratory Targeted and Sanger DNA Sequencing of Hotspot Mutation, Copy Number Determination, Transcriptome Fingerprinting Analysis, qPCR, PCR IDH1 and TERTp, EGFRvIII Status and Western Blotting* are described in detail in the Supplementary Methods.

### Clinical patient’s features and primary cell culture, population analysis and cloning

GBM tissue samples and signed informed consents were collected according to the ethical guidelines of the 2013 Declaration of Helsinki at IRCCS National Neurologic Institute “C. Besta” (Prot. 61) and classified according to the WHO guidelines. Primary tumor cells from post-surgery GBM specimens were plated at clonal density in NeuroCult NS-A medium alone (Stemcell Technologies) (I-GSCs) or containing 20 ng/ml of EGF and FGF-2 (Peprotech) (D-GSCs) [14, 27]. Patient’s data together with localization of tumors are reported in Table 1. Population, clonogenic and differentiation analyses were performed as in [14, 27, 50]. In details, to analyze D- and I-GSCs’ proliferation potential 200,000 viable cells were plated in growth medium with and without GFs (0 DIV). At each subculture passage, the total number of viable cells were counted, and 200,000 cells were replated under the same conditions. The same procedure was repeated for up to 10 subculture passages. The self-renewal index has been assessed by means of clonogenic assays. In brief, individual, D- and I-GSCs clones (spheres) from various passages were mechanically dissociated, and single cells were plated in growth medium with and without GFs at clonal density and the number of secondary spheres generated from each primary sphere was counted after 7**–**12 days. Cell line authenticity was last tested in January 2021 using CNV profiling. Table 2.

**Table 1:** Characteristics of patients and samples involved in the study

***ID SAMPLE***	***Gender***	***Clinical Age***	***Clinical IDH1***	***EGFRvIII***	***TERT PROMOTER***	***Clinical Pathology***
Patient #1	M	79	WT	NO	C228T	GBM
Patient #2	M	76	WT	NO	C228T	GBM
Patient #8	M	76	WT	YES	C228T	GBM
Patient #9	M	79	WT	YES	C228T	GBM
Patient #14	F	80	WT	NO	C228T	GBM
Patient #11	M	71	WT	NO	C228T	GBM
Patient #6	F	52	WT	NO	C250T	GBM
Patient #12	M	43	WT	NO	C228T	GBM
Patient #7	F	44	WT	NO	C228T	GBM -GLIOSARCOMA
Patient #13	M	42	WT	NO	C228T	GBM
Patient #16	M	70	WT	NO	C228T	GBM
Patient #17	F	72	WT	NO	C250T	GBM
Patient #3	M	62	WT	NO	C228T	GBM
Patient #5	F	56	WT	NO	C228T	GBM
Patient #10	F	73	WT	YES	C228T	GBM
Patient #15	M	62	WT	NO	C228T	GBM

**Table 2:** Key resources table

REAGENT or RESOURCE	SOURCE	IDENTIFIER
**Antibodies**		
Mouse monoclonal anti-Human Nuclei	Merck - Millipore	Cat# MAB1281; RRID: AB_94090
Rabbit polyclonal anti Laminin	Merck – Millipore (Sigma Aldrich)	Cat# L9393; RRID: AB_477163
Goat polyclonal anti EphA2	R&D System	Cat# AF3035; RRID: AB_2277943
Anti-Ki-67 antibody	Millipore	Cat# AB9260; RRID: AB_2142366
Rabbit polyclonal anti Wnt5a	LS Biosciences	Cat# LS-C160634; RRID: AB_2736865
Mouse monoclonal anti BMPR 1b	R&D System	Cat# MAB5051; RRID: AB_2064101
Rabbit polyclonal Glial Fibrillary Acidic Protein (GFAP)	Agilent	Cat# Z0334; RRID: AB_10013382
Mouse Anti-Galactocerebroside C (GalC)	Millipore	Cat# MAB342; RRID: AB_94857
Purified anti-Tubulin beta 3 (TUBB3)	Biolegend	Cat# 801201; RRID: AB_2313773
Goat anti mouse AlexaFluor488	Thermo Fisher	Cat#; RRID:AB_2534069
Goat anti mouse AlexaFluor546	Thermo Fisher	Cat# A11003; RRID:AB_141370
Goat anti rabbit AlexaFluor488	Thermo Fisher	Cat# A11008; RRID:AB_143165
Donkey anti goat AlexaFluor488	Thermo Fisher	Cat# A11055; RRID:AB_2534102
Goat anti rabbit AlexaFluor546	Thermo Fisher	Cat# A11010; RRID:AB_2534077
**Experimental Models: Organisms/Strains**		
Scid-Beige Mouse: CB17.Cg-Prkdc^scid^Lyst^bg^-J/Crl *Mus musculus*	Charles River	Cat# CRL:250; RRID:IMSR_ CRL:250
**Software and Algorithms**		
AxioVision Imaging System	Zeiss	RRID:SCR_002677
NIS-Elements	Nikon	RRID:SCR_014329
GraphPad Prism	http://www.graphpad.com/	RRID:SCR_002798
Integrative genomic viewer (IGV)	http://software.broadinstitute.org/software/igv/	RRID:SCR_011793
R Project for Statistical Computing	https://www.r-project.org/	RRID:SCR_001905
PARTEK GENOMICS SUITE	https://www.partek.com/	RRID:SCR_011860
ARRAYEXPRESS REPOSITORY	https://www.bioconductor.org/packages/release/bioc/html/ArrayExpress.html	RRID:SCR_000120
VARSCAN	http://tvap.genome.wustl.edu/tools/varscan/	RRID:SCR_006849
ANNOVAR	http://www.openbioinformatics.org/annovar/	RRID:SCR_012821
GATK	https://software.broadinstitute.org/gatk/	RRID:SCR_001876
MutationTaster	http://www.mutationtaster.org/	RRID:SCR_010777
PolyPhen: Polymorphism Phenotyping	http://genetics.bwh.harvard.edu/pph2/	RRID:SCR_013189
PROVEAN	http://provean.jcvi.org/	RRID:SCR_002182
SIFT	https://sift.bii.a-star.edu.sg/	RRID:SCR_012813
Circos	http://circos.ca/	RRID:SCR_011798
Ingenuity Pathway Analysis	http://www.ingenuity.com/products/pathways_analysis.html/	RRID:SCR_008653
GISTIC	http://www.mmnt.net/db/0/0/ftp-genome.wi.mit.edu/distribution/GISTIC2.0/	RRID:SCR_000151
SAMTOOLS	http://www.htslib.org/	RRID:SCR_002105
CustomCDF	http://brainarray.mbni.med.umich.edu/Brainarray/Database/CustomCDF/genomic_curated_CDF.asp/	RRID:SCR_018527
Entrez Gene	http://www.ncbi.nlm.nih.gov/gene/	RRID:SCR_002473
BaseSpace	https://basespace.illumina.com/home/sequence/	RRID:SCR_011881
CHROMAS	https://technelysium.com.au/wp/chromas/	RRID:SCR_000598
QIAXCEL	QIAGEN	RRID:SCR_018624

### Invasion assays

I- and D-GSCs’ migration capacity was evaluated by invasion assays (Corning Costar) [27]. The upper side of the filter was coated with Cultrex (Trevigen) and 2 × 10^5^ cells were seeded. Two weeks after plating, cells on the upper side were mechanically removed, and those migrated onto the lower side were fixed and stained. Wnt5a manipulation was performed by rhWnt5a (2μg/ml; R&D System), rhWnt3a (2μg/ml; R&D System) and rhSFRPs (0.3uM; R&D System) proteins administration. Enhancement of Wnt5a expression was accomplished by Wnt5a lentiviral-mediated overexpression [27].

### In vivo studies

Animals were housed at University of Milan-Bicocca and procedures were performed in accordance with the Guidelines for the Care and Use of Laboratory Animals and animal experimental protocols approved by the Ministry of Health (prog. N°7/2010 and 7/2013). In order to minimize any suffering of the animals, anesthesia and analgesics were used when appropriate. Tumorigenicity was determined by stereotactic injection of I- and D-GSCs cells into the right striatum of immunocompromised *SCID* mice (Charles River Lab) [14, 27]. Mice were then sacrificed at different endpoints, comprised between 4 and 12 weeks post-transplantation, according to the subtype of the GSCs line injected. Immunohistochemistry was performed on 15 μm-thick cryostat sections [14, 27]. Infusion of PepA and ephrinA1-Fc as single or combinatorial treatment into the tumor was obtained by means of osmotic mini-pumps for up to 2 weeks. To overcome the delivery limitations in the central nervous system we routinely utilize these local delivery systems capable of sustained release through surgical implantation within the tumor site, thus mimicking the intracranial convention enhanced delivery (CED) used in patients [51]. Briefly, the catheter of osmotic mini-pumps (Durect Corporation) was placed through the same burr hole into the mice striatum after orthotopic injection of both GSCs. 100 ul of PBS alone (Control), containing PepA (100 μg) and ephrinA1-Fc (30 μg) alone or in combination were placed in the reservoire of the Alzet brain infusion kit III (Durect Corporation) and infused for 14 days (0.25ul/h).

### Survival analysis

To evaluate the relationship between the level of *WNT5A* and *EPHA2* and patients’ outcome, 236 IDH1 wild-type GBM patients were selected in the TCGA dataset and mRNA expression data and corresponding clinical information downloaded from https://xenabrowser.net/datapages/. Optimal cutoff between high and low mRNA expression groups were determined through the R package “survminer”. TCGA-PRO, TCGA-CL and TCGA-MES subtypes were then selected, obtaining two subgroups: *WNT5A*^High^*EPHA2*^Low^ (*n* = 21) and *WNT5A*^Low^*EPHA2*^High^ (*n* = 70). Survival curves were evaluated using GraphPad Prism v.7.0 software by Kaplan-Meier method and overall comparisons performed by Log-rank test considering *P*-values< 0.05 significant.

### Statistical analysis

For in vitro studies, statistical tests were performed using R and GraphPad Prism v7.0 software and apposite test selected according to the variance and distribution of data. Differential gene expression from microarray data was assessed by the implementation of the ANOVA test available in Partek Genomic Suite 6.6 with Benjamini-Hochberg False Discovery Rate (FDR; *q-value*) < 0.05. Growth curves were analyzed with hierarchical linear models for repeated measurements to assess trends over time [52, 53]. Log-transformed cell number was used as outcome. A spatial power correlation type was used to account for unequally spaced time occasions during the experiments [52]. *P*-values < 0.05 were considered statistically significant. All analyses were performed using SAS Statistical Package Release 9.4 (SAS Institute, Cary, NC, USA). Association of copy number alterations or mutations was determined by comparing each subgroup with a two-tailed Fisher’s exact test. In vivo comparisons between I- and D-GSCs-injected mice or control and treated mice were carried out by a hierarchical linear model for repeated measurements [27] and Dunnett’s multiple comparison test. Survival curves were estimated by GraphPad Prism v7.0 software by Kaplan–Meier method and the distribution of survival were compared by the Log-rank and GBW tests. *P*-value < 0.05 was considered significant.

## Results

### GF-independent GSCs are an intrinsic Wnt5a^High^/EphA2^Low^ invasive subset within GBM

**Fig. 1:**
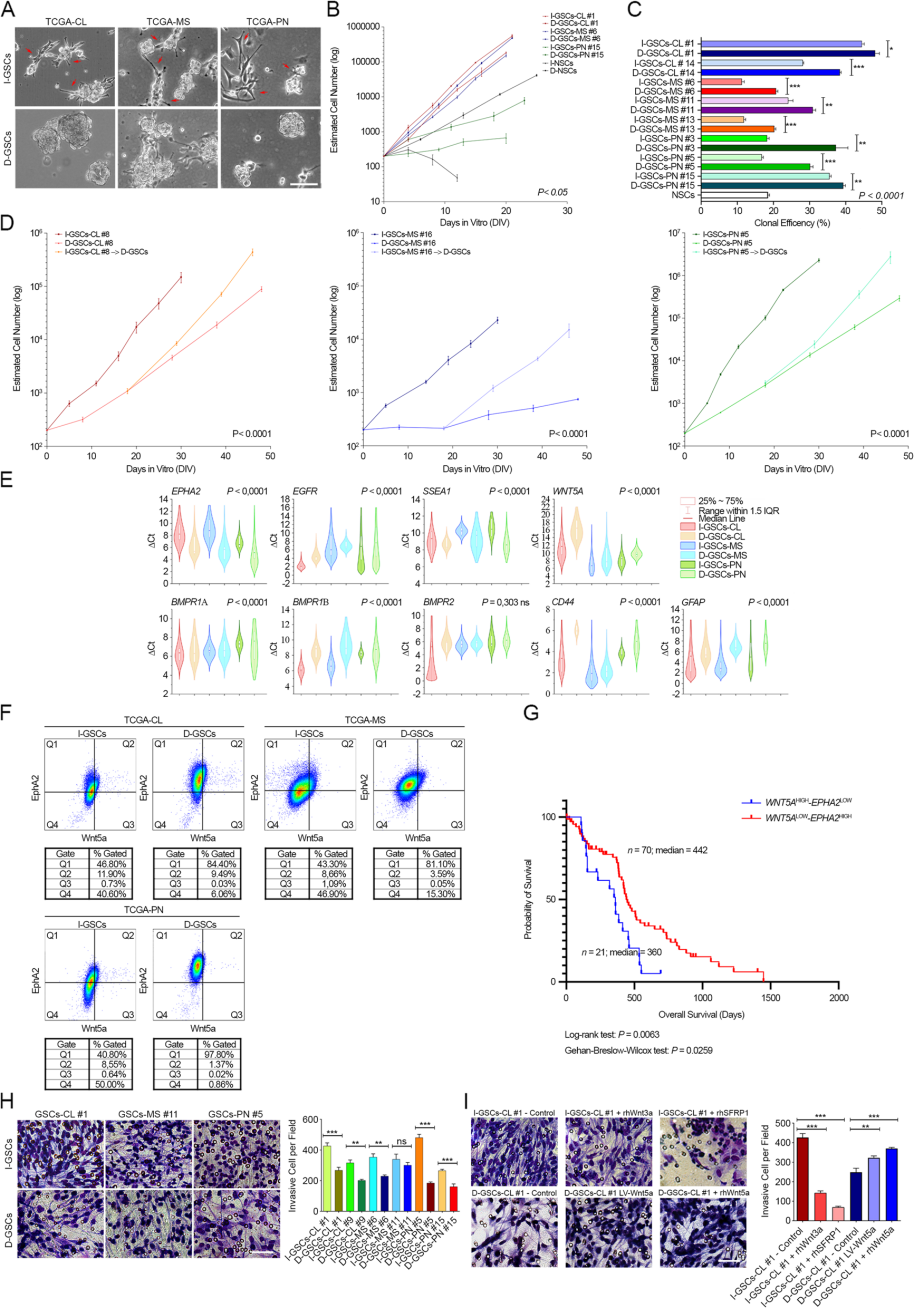
I-GSCs can be isolated from GBM surgery specimens in the absence of mitogenic stimulation. **A**. GFs-independent (I-GSCs) (top) and dependent GSCs (D-GSCs) (bottom) can be either isolated from the very same patient’s tissue across subtypes, with the former exhibiting many adhesion-related protrusions (arrowheads) and the latter typical rounded morphology. **B**-**C**. Significant differences in the expansion rate (**B**) and self-renewal potential (**C**) between I- and D-GSCs across subtypes, with the former comprising slower-dividing GSCs with a lower clonogenicity. *P < 0.05 I-GSCs vs. D-GSCs, hierarchical linear model for repeated measurements and ***P < 0.001, **P < 0.01, *P < 0.05 and *P* < 0.0001 I-GSCs vs. D-GSCs, one-way Student’s *t*-test in **B** and **C**, respectively. Lines I-GSCs and D-GSCs #1 (TCGA-CL GSCs, red), #6 (TCGA-MS, blue) and #15 (TCGA-PN, green) are shown as representative examples in B. Data are mean ± SD (B) and mean ± SEM (C) (*n* = 3). **D**. When exposed to mitogens, I-GSCs’ proliferation closely mirrors that one of D-GSCs, regardless of subtype (TCGA-CL GSCs, right; TCGA-MS GSCs, middle; TCGA-PN GSCs, right). ***P < 0.001 I-GSCs vs. D-GSCs, hierarchical linear model for repeated measurements. Data are mean ± SD (*n* = 3). **E**. Violin plot displaying the different enrichment of genes associated to stemness, differentiation and invasion in I-GSCs vs. D-GSCs across subtypes, as detected by qPCR. *P*-values are from Kruskal-Wallis test. **F**. Dot plots showing flow cytometric analysis confirming the enrichment of Wnt5a in I-GSCs across subtypes when compared to D-GSCs, shown to upregulate EphA2. Lines I-GSCs and D-GSCs #1 (TCGA-CL GSCs), #5 (TCGA-PN GSCs) and #11 (TCGA-MS GSCs) are shown as representative examples (*n* = 3). **G**. High level of *WNT5A* combined with low *EPHA2* expression is associated with lower GBM patients’ survival according to TCGA public dataset (*P* = 0.0063 and *P* = 0.0259; *n =* 91, Log-rank and Gehan-Breslow-Wilcoxon test), as depicted by Kaplan-Meier plots. **H**-**I**. In vitro migration assay showing that, irrespective of the molecular subtype, I-GSCs migrate and invade more efficiently than their D-GSCs counterpart (**H**). **I** Blockade of Wnt5a signaling by Wnt5a-endogenous antagonists (rhWnt3a; middle and rhSFRP1; right) lessens I-GSCs’ exacerbated invasiveness (top), whereas enhancement of Wnt5a expression in D-GSCs by stable lentiviral-mediated overexpression (LV-Wnt5a; middle) or by exposure to rhWnt5a (right) elicits cell migration (bottom). *Bars* in **A**, **H**-**I**, 100um, 50um. Quantification in **H**-**I** is shown as mean ± SEM. ***P < 0.001, **P < 0.01, ns not significant, one-way Student’s *t*-test and ANOVA Tukey’s multiple comparison test

To investigate as to whether GF-independent GSCs (I-GSCs) might embody an intrinsic component of the tumor itself, acutely isolated cells from IDH1-wild-type GBM specimen were plated at clonal density in serum-free medium, either in the presence of EGF and FGF2 [14, 49] or avoiding the classical mitogenic stimulation. Following exposure to GFs, typical neurospheres formed in culture and, even in the mitogen-free cultures, primary neurospheres displaying protrusion and elongation of cell shape could be detected (Fig. 1A). Interestingly, when compared to their cognate cells isolated in the presence of GFs (D-GSCs), I-GSCs displayed a peculiar functional phenotype, regardless of the subtype, i.e. TCGA-CL, TCGA-MES and TCGA-PRO [22, 54, 55]. As clearly shown in Fig. 1B-C, I-GSCs’ global growth trend (Fig. 1B and Supplementary Fig.S1A) and clonal efficiency (Fig. 1C) was somewhat lower than that of their matched D-GSCs. Upon growth factors removal, human neural stem cells (NSCs), used as negative control, were confirmed to die rapidly (Fig. 1B). Remarkably, when cultured in the presence of mitogens I-GSCs cells acquired the typical growth rate of their siblings D-GSCs (Fig. 1D). The size of neurospheres generated in mitogen-free cultures appeared smaller than that of D-GSCs, suggesting differences in the cell cycle length. This was demonstrated by the definition of I- and D-GSCs’ cell cycle signature. Yet, the former tends to display a higher percentage of cells gated in G0/G1 phase and a lesser percentage of cells gated in S phase as compared to the latter as a whole (Supplementary Fig.S1B).

Regardless of the subtype tested, I-GSCs seemed to display a more “astrocyte-like” phenotype, with a significant increase in the frequency of the astroglial differentiation marker GFAP but not in neuronal or oligodendroglial ones (Fig. 1E and Supplementary Fig.S1C). Yet, the more mature makeup of these cells, versus their GF-dependent counterpart, emerged to be sustained by a relative lower expression of markers associated to GSCs state in GBM, including SSEA-1 and EphA2 (Fig. 1E-F and Supplementary Fig.S1D). Meanwhile, I-GSCs upregulated the tissue invasiveness mediator Wnt5a, which has a key role in GSCs dispersion [27, 33], CD44 and Bone Morphogenetic Protein Receptors (BMPRs) [56] (Fig. 1E-F and Supplementary Fig.S1E). Both GSCs populations inherently displayed a different pattern of EphA2 and Wnt5a expression across subtypes [27, 32]. In any case, EphA2 and Wnt5a proteins were infrequently co-expressed. Strikingly, the Wnt5a^High^/EphA2^Low^ profile was shown to be a predictor of poor prognosis in the TCGA dataset (Fig. 1G).

We next assessed whether the highest level of Wnt5a in I-GSCs was related to their invasive potential observing that these cells, once more irrespective of the subtype, infiltrated more efficiently than their cognate D-GSCs (Fig. 1H). A key role for Wnt5a in modulating GSCs and even NSCs ability to extensively infiltrate was also confirmed (Fig. 1I and Supplementary Fig.S1F) [27, 33].

Altogether, these data report the identification and the in vitro characterization of a subset of mitogen-independent GSCs isolated from patient’s tumor specimens, by exploiting their inherent ability to self-maintain and to infiltrate and the unique aggressive Wnt5a^High^/EphA2^Low^ profile.

### I-GSCs establish tumors in vivo endowed with exacerbated lethality and intracranial invasion

**Fig. 2:**
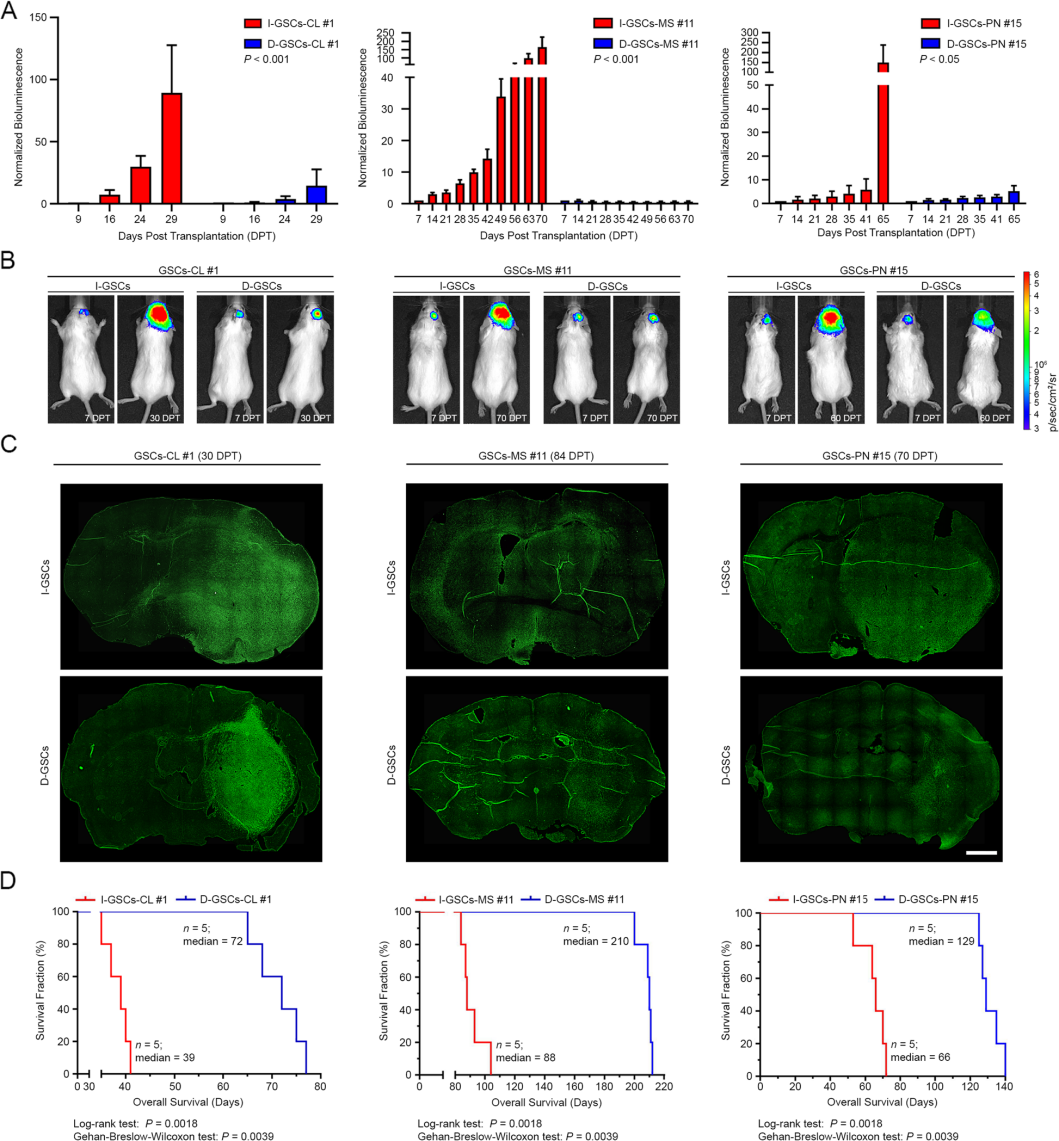
I-GSCs are endowed in vivo with enhanced tumorigenic ability. **A**. GBM xenografts derived from I-GSCs display higher growth than those generated by D-GSCs sibling regardless of the transcriptional subtype, as depicted by quantitative time-course bioluminescence analysis. Data are mean ± SEM. *P*-values are from hierarchical linear model; *n* = 5 mice/group. **B**. Mice carrying luc-I- and D-GSCs cells are imaged from 7 days post-transplantation (DPT) to the endpoint. **C**. Serial immunohistochemical reconstructions confirming that I-GSCs give rise to more extended and invasive tumors than those from D-GSCs injection. *Bar*, 1 mm. **D**. Kaplan-Meier plot of survival demonstrating that animals receiving I-GSCs die much earlier than those implanted with their sibling D-GSCs. *P*-values are from Log-rank and Gehan-Breslow-Wilcoxon test

To verify as to whether mitogen withdrawal might affect also the overall in vivo tumorigenic and invasive capacity of GSCs, I- and D-GSCs were infused orthotopically into immunocompromised *SCID* mice [14, 27]. As expected, upon intracranial transplantation, both GSCs subpopulations were shown to give rise to prototypical human GBM. Strikingly, Wnt5a^High^/EphA2^Low^ I-GSCs’ tumorigenicity was exacerbated and so did their lethal capacity, irrespectively of the subtype (Fig. 2 and Supplementary Fig.S2). We found that, as early as 30–80 days post-transplantation (DPT), depending on the median end-stage peculiar of each GSCs line, tumors from I-GSCs-bearing mice were much more expanded and able to very rapidly spread all throughout the brain parenchyma, as compared to those from D-GSCs-injected mice (Fig. 2A-C and Supplementary Fig.S2A-B). Yet, the rate of proliferation was higher in I-GSCs-derived tumors and so did the rate of vascularization, with a significant increased vessel density ranging from 4 to 10-fold with respect to those from D-GSCs-carrying mice (Supplementary Fig.S2C). Consistently, mice receiving I-GSCs exhibited more lethal tumors with an overall survival that was more than two times shorter than those of animal carrying D-GSCs-derived tumors. Yet, a median survival window of only 39, 66 and 88 days was peculiar of TCGA-CL, TCGA-PRO and TCGA-MES I-GSCs-receiving mice, respectively, whereas D-GSCs-implanted counterpart survived for 72, 129 and 210 days (Kaplan-Meier survival analysis; *P* < 0.01, Log-rank and Gehan-Breslow-Wilcoxon tests, *n* = 5 mice/group) (Fig. 2D).

Data so far demonstrate that isolating GSCs from patient’s tumor specimens under more physiological condition, i.e. avoiding artificial GF-stimulation, exposes one of the most dangerous “hostile” traits of GBM, that is its tumorigenicity and invasiveness, as exacerbated.

### I-GSCs faithfully resemble the tissue of origin and display a distinctive “motile” transcriptomic fingerprint

**Fig. 3:**
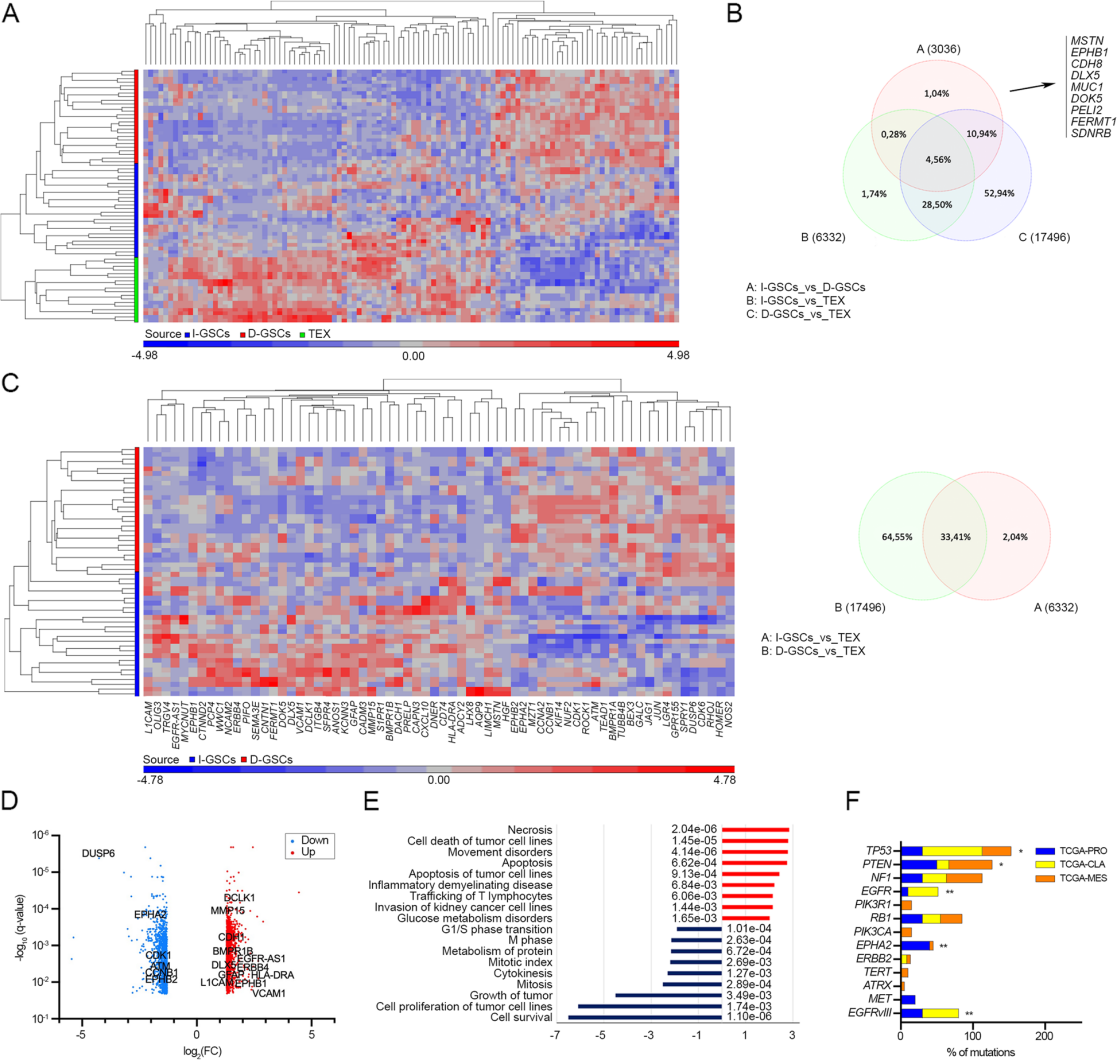
I-GSCs’ transcriptional profile is defined by migration and invasiveness. **A**-**B** Unsupervised hierarchical clustering analysis based on global gene expression profile of nine I-GSCs lines (blue), D-GSCs (red) counterpart and their GBM tissues (TEX; green) reporting a matching transcriptional signature between I-GSCs and tissue samples. Samples are coded by color. A dual-color code represents genes over- (red) and under-represented (blue), respectively (**A**). **B** Venn diagram confirming the exclusive over-expression in I-GSCs of genes mainly involved in cell migration (FDR < 0.05 and FC = 2). **C**. Hierarchical clustering using 66 differentially expressed genes when comparing I-GSCs to D-GSCs (left). Higher overlap of genes between I-GSCs and their original GBM tissue as compared to D-GSCs versus the same tissue, as reported by Venn diagram (right). **D**. Volcano plots based on expression data showing the higher infiltrative and mature, “astrocyte-like” profile of I-GSCs vs D-GSCs. Significant hits are depicted in red and blue. The top candidates are labelled. **E**. When compared to their D-GSCs siblings, I-GSCs’ overexpress transcripts belonging to biological functions as cell death, invasion and inflammatory/immune response, whereas downregulated mRNAs are mainly involved in cell cycle, cell proliferation and survival processes. Red and blue bars count for up- and downregulated genes, respectively. **F**. Distribution of the frequently mutated genes in GBM across subtype in both GSCs population and their tissue. **P < 0.01, *P < 0.05, Fisher’s exact test

To pinpoint the inherent GSCs’ critical regulators, with particular emphasis on those involved in spreading and tumorigenic potential, we next carried out a side-by-side comparative transcriptomic, genomic and genetic analysis of I- and D-GSCs as well as their tissue of origin. As shown in Fig. 3A, hierarchical cluster analysis of global gene expression profiles clearly distinguished GBM tissues and both GSCs subpopulations, regardless of the subtypes. Yet, a similar transcriptional signature was retrieved in GBM patient’s specimens and I-GSCs, suggesting that these cells faithfully resembled the functional characteristics of the tissue of origin (Fig. 3A-B and Supplemental Table S5) [57]. Remarkably, a distinctive expression program emerged for the GF-independent GSCs, which clearly underlined enrichment for genes controlling extracellular matrix (ECM) remodeling, cell migration and metastasis (*CTNND2*, *ANOS1*, *ENPP2*, *MMP15*, *DCLK1*, *ITGB4*, *CDH1*, *EPHB1*, *BCAN*, *PRELP*, *CXCL10, EGFR-AS1, AQP9*), calcium ion binding (*PCP4*, *ADCY2*, *EDNRB*, *PLSCR4*, *ANXA8L1*) cell focal adhesion (*CNTN1, MID1*, *CADM3*, *NCAM*, *L1CAM*) and angiogenesis (*VCAM*, *EDNRB*) (Fig. 3C-D and Supplemental Table S6). High level of monocyte chemotactic factor (*CCL*) and of genes associated with coagulation and immune/complement responses (*CD74, HLA-DRA, TRGV4, IGKV1–6*) also indicates a pro-inflammatory state. Several genes were almost confirmed to regulate differentiation (*GFAP*, *OLIG3*, *BMPR1B*, *MYCNUT*, *DLX5*, *DNER*) and to encode for RTK activity, critical in the oncogenesis of GBM, including *NTRK, REPS2, ERBB4* and *DOK5*, confirming an “hybrid” astrocyte-like/mesenchymal-like (AC-like/MES-like) state within this subset of GSCs [58]. Nevertheless, the GF-dependent counterpart was defined by genes controlling mitotic cell cycle and cytokinesis-associated genes (*CCNB1*, *ATM*, *CDKN3*, *KIF14, KIF20B)*, cell growth, proliferation, cycling and stemness (*CDK1*, *DUSP6*, *LAMC1*, *MAPK10*, *YES1, EPHA2*, *EPHB2*) (Fig. 3C-D). These data were also confirmed when the emerging profile of I- and D-GSCs across subtypes was compared to each other (Supplementary Fig.S3A-B). Consistently, significant enhancements were found in the expression of selective biological signaling including apoptosis and necrosis, cellular invasion and immune cell trafficking in the GF-independent cells versus D-GSCs counterpart (Fig. 3E, Supplementary Fig.S3C and Supplementary Table S1), which was confirmed to embody the subset of cycling and proliferating cells.

When the distribution of somatic mutations, including SNVs and indels, and copy number changes were evaluated focal subtype-specific aberrations typically associated to GBM were similarly identified between the two culture conditions [22, 54]. Yet, mutation-calling analysis revealed that samples from TCGA-CL subtype harboured mutations in *EGFR* and *TP5*3 genes*,* whereas the higher rate of somatic variant mutations in *NF1*, *PTEN*, *EPHA2* and *MET* genes occurred predominantly in TCGA-MES and TCGA-PRO cases, respectively (Fig. 3F and Supplementary Tables S2–3). Several of the typical well-defined GBM-related, arm-level changes were also found, as emerged from analysis of copy number variations. Well in line with the transcriptomic fingerprint (Fig. 3C), focal amplifications typical of D-GSCs cells, regardless of the subtype, were detected at 5q34 (*q-*value = 0.037, two-sided Fisher’s exact test) (*TERT, CCNB1*) and 1p36.13 (*q*-value = 0.011) (*LAMC1*, *KIF14*, *EPHA2*, *EPHB2*), whereas focal amplification at 9q31.1 (*q*-value = 0.015) (*INVS*, *MURC*) was specific for the I-GSCs counterpart (Supplementary Fig.S3D and Supplemental Table S4).

Taken together, all of these findings confirmed that the relatively in vitro slow-propagating subset of I-GSCs is defined by a peculiar “mesenchymal-like” molecular signature with specific genes controlling the enhanced invasive phenotype and tumorigenic potential, as compared to GF-dependent counterpart.

### Clinical potential of the combined modulation of Wnt5a and EphA2 activity

Having observed a unique, lethal Wnt5a^High^/EphA2^Low^ profile specific for the highly invasive I-GSCs cells as opposed to that Wnt5a^Low^/EphA2^High^ of the proliferative D-GSCs siblings, to address the therapeutically cogent issue of how to tackle GSCs and along the line of developing combinatorial approaches, we next explored the combined effects of modulating Wnt5a and EphA2 in vitro and in vivo on both GSCs subpopulations.

**Fig. 4:**
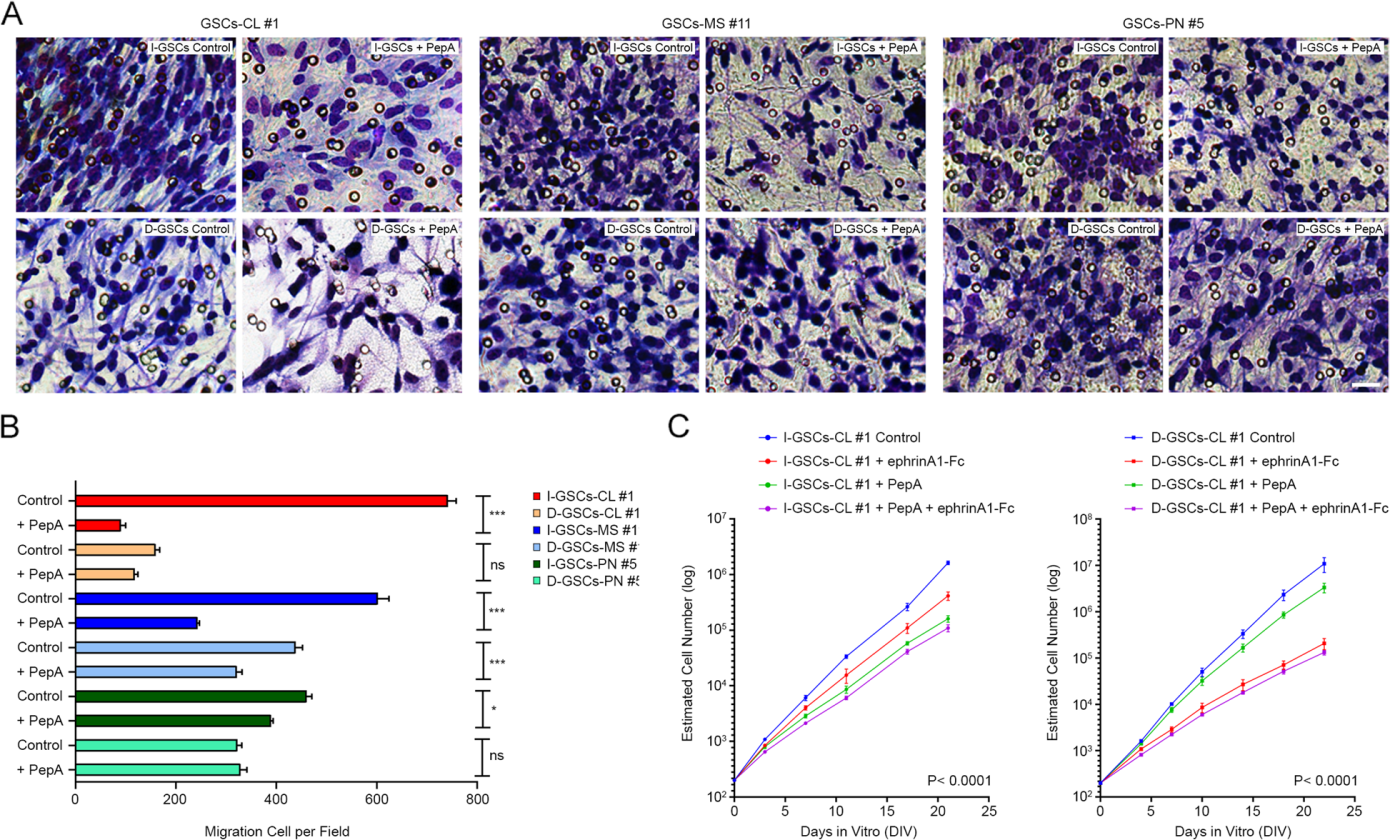
Ex vivo effects of PepA and ephrinA1-Fc treatments on GSCs. **A** Ex vivo treatment of GSCs with exogenous PepA hinders invasiveness of either I- (top) or D-GSCs (bottom) regardless of subtype. *Bars*, 50um Quantification is shown in **B** as mean ± SEM. ***P < 0.001, *P < 0.05, ns, not significant, one-way Student’s *t*-test; *n* = 3. **C**. Exposure of I- (left) and D-GSCs (right) to PepA (green line), ephrinA1-Fc (red line) or a combination of both (purple line) lessens steady growth of both GSCs to nearly half than in control GSCs, showing a clear additive effect of the two molecules. Data are mean ± SD. ***P < 0.001 I-GSCs vs. D-GSCs, hierarchical linear model for repeated measurements; *n* = 3

As depicted by invasion assay in Fig. 4A-B, a counteraction of Wnt5a activity by the previously described Wnt5a antagonistic hexapeptide PepA [27] significantly hindered in vitro invasiveness of I-GSCs and, to a lesser extent, of D-GSCs cells. Furthermore, in vitro administration of ephrinA1-Fc alone, the EphA2 cognate ligand [59], reduced more effectively D-GSCs’ growth potential, the major site of EphA2 overexpression, as compared to I-GSCs counterpart (Fig. 4C). Meanwhile, the highest suppressive effect on GSCs proliferation ability of PepA as single-agent was observed in the latter, upregulating Wnt5a. Remarkably, as clearly outlined in Fig. 4C, when both GSCs were exposed simultaneously to ephrinA1-Fc and PepA, the combination of the two molecules treatment was observed superior to the single treatments alone with an additive effect in terms of lessening GSCs’ proliferation.

**Fig. 5:**
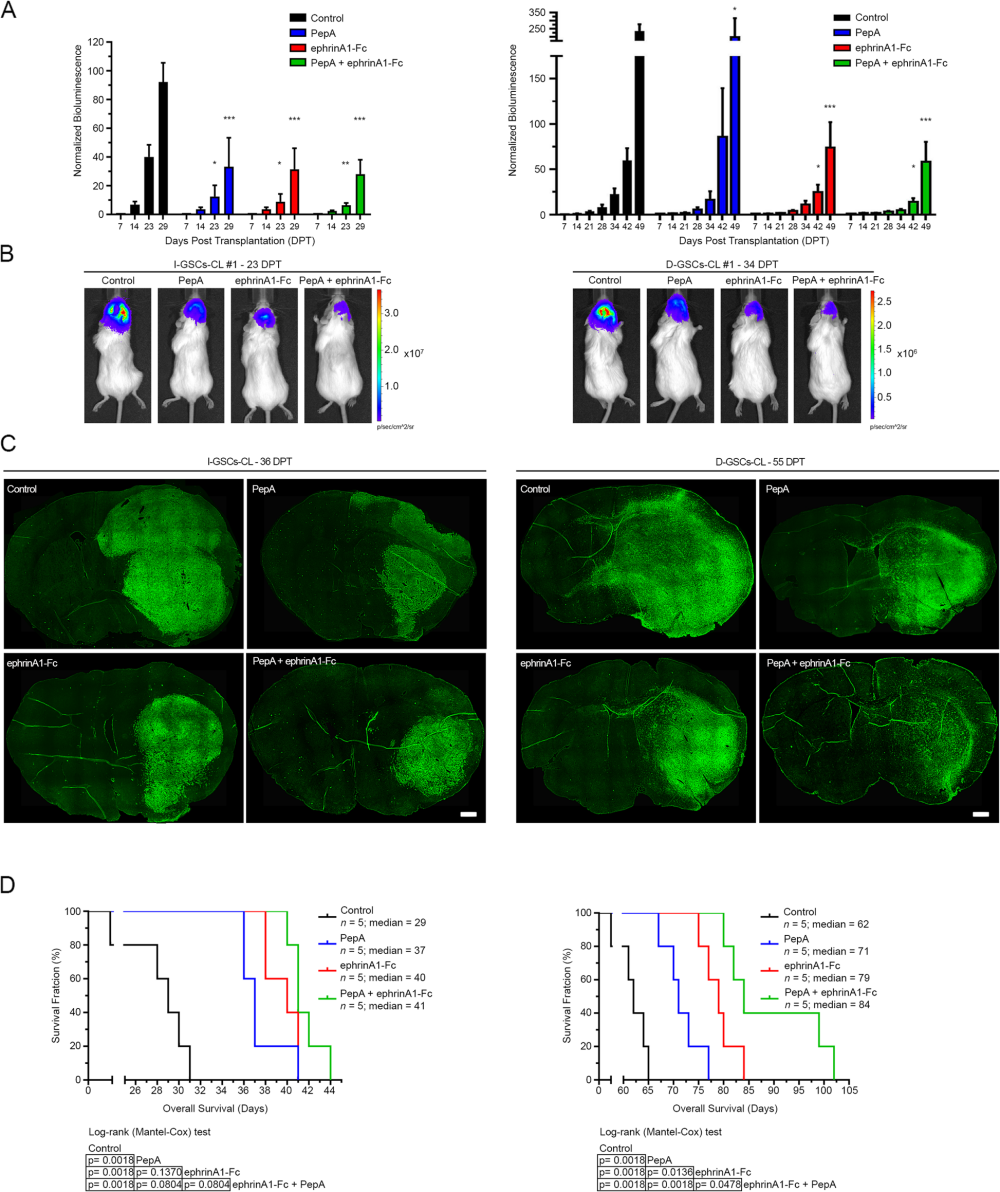
A combinatorial PepA/ephrinA1-Fc-manipulation approach impairs tumorigenicity and invasiveness of GSCs cells. **A**. Quantitative analysis of luc-GSCs signal showing that both PepA and ephrinA1-Fc hinder I-GSCs-derived tumors’ growth (left) and, to a lesser extent, of their D-GSCs counterpart (right) and that the combined use of the two molecules has an additive effect. Data are mean ± SEM. ***P < 0.001, *P < 0.05, Dunnett’s multiple comparison test; *n* = 5 mice. **B**. Imaging of luciferase-tagged I- and D-GSCs injected into the brain of *Scid/bg* mice showing that after 23 and 34 days, respectively, untreated mice develop larger and spreaded tumors than PepA and ephrinA1-Fc-infused mice. Tumor growth is markedly inhibited with the combination of both molecules. **C**. Brain sections confirming that tumors from mice carrying I-GSCs or D-GSCs cells and infused with vehicle proliferated and spread through the brain parenchyma more than those infused either with PepA or ephrinA1-Fc, being the combination of both even more efficacious in reducing cell proliferation and invasiveness. *Bar*, 1 mm. **D**. Survival of mice harboring I-GSCs (left) and D-GSCs-tumors (right) is significantly enhanced when infused with PepA (blue bar) and ephrinA1-Fc (red bar) alone, or even more with the combination of the two molecules (green bar). A league table of comparison by Log-rank is shown; *n* = 5 mice

Next, we verified whether PepA infused in combination with ephrinA1-Fc into the brain of GBM orthotopic xenografts for 14 days by means of osmotic mini-pumps further reduced GSCs’ in vivo invasive and tumorigenic ability*,* as compared to the single treatments alone. Consistent with the in vitro observation, PepA and ephrinA1-Fc treatment alone significantly inhibited D-GSCs’ tumorigenicity, while a more significant suppressive effect of the same single-agent on I-GSCs’ growth and invasiveness were observed (Fig. 5A-C and Supplementary Fig.S4). Remarkably, the combination of PepA with ephrinA1-Fc treatment on both GSCs cells-derived tumors was superior to the single treatments alone in terms of suppressing the growth and the capacity for brain dispersal (Fig. 5A-C and Supplementary Fig.S4). Consistently, Kaplan-Meier survival analysis reported that mice receiving PepA and ephrinA1-Fc as single-agent have a significant longer life span than mice treated with vehicle (*P* < 0.0001, Log-rank and GBW tests, *n* = 5 mice/group) (Fig. 5D). Most important, intracranial simultaneous administration of the two molecules under putative therapeutic conditions, was able to hinder either tumor propagating ability (extending the overall survival from 29 and 62 days to 41 and 84 days in I- and D-GSCs controls vs. treated mice, respectively; *P* < 0.0001, Log-rank and GBW tests, *n* = 5 mice/group) or invasiveness in an additive manner and, importantly, without substantial cytotoxic effects.

These data show that Wnt5a antagonistic peptide PepA in combination with ephrinA1-Fc is able to hinder in vivo GSCs’ tumorigenicity and invasiveness in an additive manner and, as such, suggest a new potential combinatorial therapy against GSCs, under experimental settings that are conducive to clinical applications.

## Discussion

Two decades have elapsed from the initial discovery that GBMs embody cells endowed with tumor-initiating ability and all of the functional features that define stem cells of the CNS. Such GBM stem-like cells (GSCs) have now emerged as the most critical cellular target in the therapeutics of glioblastomas. In view of the recent stratification of GBMs in different subtypes and cellular programs, this has stressed the necessity to fully define the nature, functional and physio-pathological nuances and heterogeneity of GSCs. Here we show how a pool of true GSCs with the ability to perpetuate and expand in the absence of exogenous mitogens (GFs-independent GSCs; I-GSCs) is found in all GBM subtypes. Such I-GSCs differ significantly from the traditional mitogen-dependent GSCs (D-GSCs) in both their transcriptional and functional repertoire. Of importance, I-GSCs are remarkably more aggressive than D-GSCs, in good part due to their paroxysmal invasion ability, that emerges from a hyperactive non-canonical Wnt5a signaling, associated to a lesser expression of the EphA2 receptor as compared to D-GSCs. These features help to associate I-GSCs to a specific functional and immuno-phenotype. This peculiar body of features sets the stage for a combinatorial treatment in mouse-human xenografts GBM model that significantly reduces I-GSCs tumorigenicity and increases survival under settings that portend clinical applications.

Saturating concentrations of growth factors such as EGF and FGF2 are used to culture and multiply normal human neural stem cells that require them to undergo expansive self-renewal ex vivo [42] – a technique subsequently applied to GSCs routinely [37, 38]. Yet, many cancer cells are self-sufficient in their requirement for mitogenic stimulation [39, 41], a notion extended to some I-GSCs [39, 44–46]. A decade down the road it remains unclear if such I-GSCs are present in all GBM subtypes [22, 26, 54, 55, 58]. We found that, I-GSCs cells completely self-sufficient in the activation of their own growth signaling machinery can be found in all of the glioblastoma sub-classes. In fact, we could isolate stable I-GSCs lines, in the absence of added growth factors, regardless of the surgery specimen belonging to the TCGA-PRO, TCGA-MES or TCGA-CL GBM subtype (Fig. 1A). Notably, using growth factors, we could always establish D-GSCs lines from the same specimens embodying the I-GSCs. When compared side-by-side, sibling I-GSCs and D-GSCs were found to differ significantly from each other. First and foremost, D-GSCs displayed a higher clonal efficiency and expanded faster ex-vivo (Fig. 1B-C and Supplementary Fig.S1A-B) but were much less invasive and lethal than I-GSCs in vitro or in vivo (Fig. 1H, Fig. 2 and Supplementary Fig.S2). I-GSCs always gave rise to larger GBMs in the mouse brain and spread rapidly throughout the parenchyma, with a much more significant tumorigenic and lethal capacity.

The fundamental differences in the properties of I- and D-GSCs of an identical origin provided us with the opportunity to carry out a set of differential molecular analyses aiming at identifying critical putative effectors of carcinogenicity. I- and D-GSCs cells and the tissue from which they were isolated were thus subjected to a side-by-side comparative analysis that put emphasis on detecting factors involved in intracranial spreading and tumorigenic potential. We confirmed that I-GSCs’ highly malignant behavior was dependent on the preservation of a “mitogen-independent” phenotype, underpinned by a striking pro-migratory and invasive transcriptional signature. Thus, a “astrocytes/mesenchymal-like” fingerprint was always associated to the expression of an “invasion signature” and to an obvious, paroxysmal infiltration ability in I-GSCs (Fig. 1E and Fig. 3C-E, Supplementary Fig.S1C and S3A-C). Remarkably, the pattern of gene expression of the original tumor specimens was preserved more faithfully in I-GSCs than in D-GSCs (Fig. 3A-B). This suggests that the lack of exogenous mitogens may led to the preservation of the peculiar functional phenotype that characterizes GSCs in GBMs. In fact, I-GSCs not only faithfully recapitulate the main features of the most aggressive GBM cells, such as the relatively low mitotic index ability and the high capacity for brain dispersal, but also specifically express mediators of tissue invasiveness and angiogenesis, which make these cells “primed” for in vivo tumorigenesis.

We have recently shown that overexpression of Wnt5a drives many signaling pathways regulating migration, infiltration and invasion in GSCs, thereby enhancing their lethality [27]. Here, we found that, in fact, I-GSCs express much higher levels of Wnt5a than their D-GSCs counterpart which, conversely, are a major site of expression for EphA2, a critical regulator of GSCs activity [32] (Fig. 1E-F, Fig. 3C-D and Supplementary Fig.S1D). That a Wnt5a^High^ and EphA2^Low^ fingerprint highly correlates with poor survival in GBM patients (Fig. 1G), confirmed our observations and points to the fact that this immuno-phenotype may define pools of GSCs endowed with exacerbated aggressiveness.

Tackling GSCs by simultaneously targeting multiple effectors of their aggressiveness may prove a superior therapeutic strategy. The master role played by Wnt5a and EphA2 in regulating GSCs physiology [27, 31] confirms these molecules as prominent and specific therapeutic targets. Hence, we investigated the prospective effects of manipulating Wnt5a and EphA2 activity in GSCs [27, 59], alone or in a combinatorial fashion, either ex-vivo or in vivo. We found that an anti-Wnt5a peptide and ephrinA1-Fc have the potential to effectively antagonize GSCs’ tumor propagating and invasive ability in vitro*,* substantially increasing survival in vivo (Fig. 4, Fig. 5 and Supplementary Fig.S4), when used alone. Furthermore, blocking Wnt5a and EphA2 activity with the two agents in combination, not only reduced tumorigenicity and invasiveness, in vivo*,* addictively but also prevented the recurrence of the tumor upon suspension of their intracerebral infusion after 14 days (Fig. 4, Fig. 5 and Supplementary Fig.S4)*.* This occurred in the absence of cytotoxic effects. As these experiments were conducted under pre-clinical conditions our observations are conducive to therapeutic applications in clinical settings. They may pave the way to more specific combinatorial treatments, that integrate diverse approaches aimed at simultaneously hindering the proliferation and the spreading of the GBM’s very tumor-initiating cells, i.e. GSCs.

Some final considerations that relate to the key properties and polyhedric nature of GSCs are order which bear on both our understanding of GBM physiopathology and the development of novel therapeutics. First, what is the lineage relationship, if any, between I- and D-GSCs? Both GSCs types coexist inside the same GBM, irrespective of the subtype (Fig. 1A) and I-GSCs can be converted to the D-GSCs more proliferative functional phenotype (Fig. 1D) when exposed to exogenous mitogens and the opposite phenomenon is also true (Fig. 1A and data not shown). This observation finds support in our previous findings showing that a similar situation could be observed when manipulating exposure of D-GSCs to Wnt5a [27]. This suggests that, rather than being two completely distinct GSCs populations, the I-GSCs and D-GSCs pools likely overlap, at least partially, so that for some GSCs in GBM the acquisition of a highly invasive/less proliferative behavior pertains to the presence of specific environmental cues. Whether this is a general GSCs property or an idiosyncratic feature of a specific GSCs subsets in GBMs remains to be determined. Notwithstanding, this lends to the idea that GSCs may, in fact, have the ability to “oscillate” between distinct functional states under distinct conditions – a phenomenon that we observed recently in cancer stem cells of colon carcinoma [50]. By this they would display mutating aggressivity and molecular, antigenic and functional properties, thus becoming a “moving target”, rather difficult to tackle and eradicate. If this is true, more flexible therapeutic strategies will be needed to target the different molecular and functional states that GSCs may have access to. This study exposes and defines this situation, identifies some of said functional and molecular states, thereby proposing a combinatorial treatment that antagonizes both the Wnt5a and EphA2 pathways for prospective, unconventional, combinatorial biotherapies for GBMs.

## Conclusions

In the present study, we provide the unprecedent demonstration that an “autocrine” GSCs subset (I-GSCs) is found within GBM tumors and that such cells more faithfully recapitulate than their traditional mitogen-dependent GSCs counterpart (D-GSCs) the main features of their tissues of origin, being endowed with exacerbated tumorigenicity and invasiveness. We identify a biomarker signature distinctive for such cells setting the stage for a new combinatorial strategy in mouse-human xenografts GBM model that significantly reduces GSCs tumorigenicity and increases survival under settings that portend patient-tailored clinical applications.

## Supplementary Information

**Additional file 1.**

**Additional file 2.**

**Additional file 3.**

**Additional file 4.**

**Additional file 5.**

**Additional file 6.**

**Additional file 7.**

## Data Availability

Transcriptome, Cytoscan array and Targeted sequencing raw data are available in the Arrayexpress repository under the accession codes E-MTAB-10401, E-MTAB-10400 and E-MTAB-10418, respectively.

## Abbreviations

GBM: Glioblastoma multiforme
GSCs: Glioma stem cells
I-GSCs: Growth factor independent glioma stem cells
D-GSCs: Growth factor dependent glioma stem cells
GF: growth factor
FDR: False discovery rate
Wnt5a: Wnt Family Member 5A
EphA2: Eph Receptor A2
IDH1: Isocitrate Dehydrogenase 1
NSCs: normal neural stem cells
TERT: Telomerase Reverse Transcriptase
EGFR: Epidermal Growth Factor Receptor
TCGA-CL: Classical Cancer Genome Atlas
TCGA-PRO: Proneural Cancer Genome Atlas
TCGA-MES: Mesenchymal Cancer Genome Atlas
DPT: Days post transplantation
