# Supplementary material for: Growth factor independence underpins a paroxysmal, aggressive Wnt5aHigh/EphA2Low phenotype in glioblastoma stem cells, conducive to experimental combinatorial therapy
Source: J Exp Clin Cancer Res. 2022 Apr 12;41:139. doi: 10.1186/s13046-022-02333-1 (PMC9004109; doi:10.1186/s13046-022-02333-1)
Supplement: Supplementary file 1 — Additional file 1. [file 13046_2022_2333_MOESM1_ESM.pdf]

Growth Factor Independence Underpins a Paroxysmal, Aggressive Wnt5a^{High}/EphA2^{Low} Phenotype in Glioblastoma Stem Cells, Conducive to Experimental Combinatorial Therapy

Nadia Trivieri, Alberto Visioli, Gandino Mencarelli, Maria Grazia Cariglia, Laura Marongiu, Riccardo Pracella, Fabrizio Giani, Amata Amy Soriano, Chiara Barile, Laura Cajola, Massimiliano Copetti, Orazio Palumbo, Federico Legnani, Francesco DiMeco, Leonardo Gorgoglione, Angelo L. Vescovi and Elena Binda

Supplementary information

Supplementary Method

Supplementary Figure 1-4

Supplementary Table 1-4

Supplementary Methods

Immunofluorescence I- and D-GSCs cells were seeded onto Cultrex (Trevigen)-coated glass coverslips and subjected to immunofluorescence analysis (see following section) as described earlier (1-4). Hematoxylin and Eosin (H&E) and immunofluorescence staining on human GBM xenografts was performed on OCT-embedded, 10 µm-thick cryostat sections (1-3). Images (n=10 fields/each sample) were analysed by Zeiss Axioplan2 and Nikon A1 microscopes.

Antibodies and reagents Antibodies/antisera used were: mouse anti-human Nuclei (1:100; Chemicon), rabbit anti-GFAP (1:200; Agilent), mouse anti-Galactocerebroside C (1:200; Millipore), mouse anti-Tubulin beta III (1:400; Biolegend), rabbit anti-Laminin (1:400; Millipore), goat anti-EphA2 (1:50; R&D System), rabbit anti Ki-67 (1:200; Millipore), rabbit anti-Wnt5a (1:50; LS Bioscience), mouse anti BMPR-1b (1:200; R&D System), goat anti mouse AlexaFluor488 (1:1000; Thermo Fisher), goat anti mouse AlexaFluor546 (1:1000; Thermo Fisher), donkey anti goat AlexaFluor488 (1:1000; Thermo Fisher), goat anti rabbit AlexaFluor546 (1:1000; Thermo Fisher).

Wnt5a activity was antagonized by a two week-long, intraparenchymal infusion (1) of a Wnt5a-derived hexapeptide (PepA) with the sequence Ac-Leu-Glu-Cys-Gly-Asp-Met-NH_2 (Acetyl-LECGDM-CONH2). Wnt5a-derived hexapeptide (Pep A) is a peptide derived from Box 5, a hexapeptide (tbutoxycarbonyl-Met-Asp-Gly-Cys-Glu-Leu), modified from Foxy-5, developed for use in melanoma, which is a potent, selective antagonist of Wnt5a-mediated migration and invasion of melanoma cells, both of which are essential components of the metastatic process in melanoma (5). Wnt5a-derived hexapeptide (Pep A) by inhibiting wnt5a-induced protein kinase C and Ca 2+ signaling, antagonizes the effects of Wnt5a on Glioblastoma cell migration and invasion and exerts anti-tumorigenic effects in vivo (1).

EphA2 expression and/or activity was counteracted by ephrinA1-Fc, an effective ligand of EphA2 receptor. The ephrinA1-Fc is composed by the ectodomain ephrin A1 (residues 1 to 573 of the long form of receptor) linked to the CH2 and CH3 regions of human IgG1 (starting with Glu-Pro-Lys),

with a spacer having sequence Gly-Pro-Gly insert between the ectodomain and Fc region. (6). Here, we used a mouse ephrinA1-Fc (R&D) that shares 85% and 94% aa sequence identity with human and rat ephrinA1, respectively. The inhibited efficacy of ephrin-A1 on gliomas is accompanied by the down-regulation of EphA2 and FAK (7) and in GBM CSCs ephrinA1-Fc transiently increases EphA2 tyrosine phosphorylation, in a dose-dependent manner, causing a strong and persistent downregulation of EphA2 expression (8).

Flow cytometry analysis GBM cells were collected, washed twice with PBS and then stained for 30 minutes at room temperature (RT) with zombie dye violet (1:500) (Biolegend). Subsequently, cells were incubated for 30 minutes at RT with blocking solution (PBS+2mM EDTA+ 5%FBS). Cells were then washed and stained with goat anti-EphA2 (R&D System) antibody (0.5ug/5x10^5 cells, 1h RT) and next with anti-goat AlexaFluor 647 (1:250, 1h RT, Invitrogen). After one wash with PBS cells were fixed and permeabilized with cytofix/cytoperm reagent kit (Becton Dickinson) and stained with rabbit anti-Wnt5a (ThermoFisher) antibody (1:25, 1h RT) and then with anti-rabbit AlexaFluor 488 (1:250, 1h RT), according to the manufacturer's instructions. Cells were acquired with Gallios instrument (Beckman Culter) and analysed with Flow jo X software. For cell cycle analysis, cells were collected by centrifuge, rinsed twice with phosphate buffered saline (PBS pH 7.4) and ri-collected by centrifugation. Pellets were resuspended in ice cold 70% ethanol and stored at 4°C for a minimum of 2 hours. Cells were then collected by centrifugation, rinsed twice in PBS and resuspended in 25 ug/ml propidium iodide (PI) in PBS with 20 ug/ml RNase A. Cells were stored (protected from light) at room temperature and Fluorescence (FL2) was measured using a Beckman Coulter Gallios cytometer. Cells cycle phase was then estimated using kaluza software.

Microarray analysis of gene expression Gene expression profiling was obtained by the Affymetrix GeneChip® Human Transcriptome Array 2.0 (Affymetrix) as previously described (1,3). Quality control steps and expression data analysis of .CEL files was performed using R (ver. 3.6.0) (1,3). Probes were mapped to Entrez gene ID using custom CDF files (ver. 24) for the HTA2.0 arrays from http://brainarray.mbni.med.umich.edu/Brainarray/Database/CustomCDF/CDF_download.asp.

mRNA abundance levels were analysed with Partek Genomics Suite package ver. 6.6 and *P*-values corrected by the Benjamini–Hochberg false discovery rate (FDR; *q*-values). *Q*-value <0.05 was considered significantly differentially expressed. Analysis of the biological functions, canonical pathways and regulatory networks of differently expressed mRNAs was performed by Ingenuity Pathway Analysis (IPA; Qiagen, http://www.ingenuity.com/) and R software (1,3). To identify canonical pathway and biological functions a right-tailed Fischer's exact test was performed and *P*-values were further adjusted using FDR. Cutoff for significance was set as *q*-value < 0.05, z-scores > 2 (minimum activation threshold) and z-score < -2 (minimum inhibition score) (1,3,4).

Quantitative real-time RT-PCR Total RNA was extracted using the RNeasy Mini kit (Zymo Research) with a genomic DNA removal step and cDNA obtained as earlier described (1,3,4). qPCR reactions were run in triplicate using ITaq™ Universal SYBR Green Supermix (Bio-Rad), recorded in real-time (Chromo 4 Four-Color Real-Time PCR Detector, MJ Research, Bio-Rad) and normalized to *GAPDH* as endogenous control. Gene expression profile was delineated by the Delta Ct method of relative control and mRNA levels. The relative expression level is depicted in violin plot, that is, interquartile ranges (IQRs; white bars), medians (lines on the bars), the lowest and highest values within 1.5 times IQR from the first and third quartile (lines above and below the bars) and density of values (width between curves).

Exploratory targeted DNA sequencing of hotspot mutation and data analysis Genomic DNA from GSCs lines, their GBM tissue of origin and matched peripheral blood was used for high-depth sequencing using Illumina MiSeq Platform (Illumina). Protein-coding regions of the 27 genes altered in GBM (9-11) or key in GSCs stemness and differentiation were analysed: *ATRX, KRAS, EPHA2, NRAS, IDH1, CTNNB1, PIK3CA, WNT5A, PDGFRA, PIK3R1, TERT, BRAF, EGFR, MET, CDKN2A, BMP4, BMPR1A, BMPR1B, BMPR2, MGMT, PTEN, CCND2, CDK4, RB1, ERBB2, NF1, TP53*. A Truseq Custom Amplicon Kit and AmpliSeq kit were designed by Illumina Design Studio (Illumina). Libraries were prepared from 250 ng of double stranded genomic DNA (3) and sequenced by Illumina MiSeq platform. Sequences were then demultiplexed by MiSeq Reporter software

(Illumina Inc) and results written to FASTQ files. Read mapping and variant calling analysis were performed as previously described (3,12). Somatic SNVs and indels were identified in tissue samples with matched blood DNA by integrating the results from GATK and VarScan (13,14). To filter out germline variants, nucleotide variants in samples with unavailable blood DNA were identified using GATK HaplotypeCaller corrected with a panel of normal samples as a substitute for the missing matched normal. Somatic variants were annotated using AnnoVar algorithm (15), which aggregates information from genomic and protein resources (GENECODE, UniProt, dbNSFP) with cancer (COSMIC, ClinVar) and non-cancer variant databases (dbSNP, 1000 Genomes, Kaviar, Haplotype Reference Consortium, Exome Aggregation Consortium, NHLBI Exome Variant Server). Among the annotated variants, we selected only those producing a direct effect on the protein sequence (missense, truncating, stoploss, splicing variants, frameshift, and in-frame indels). Variants reported in the non-cancer databases with a minor allele frequency ≥ 0.05 were classified as germline polymorphisms and then excluded. The functional effect of missense SNVs and in-frame indels was determined using multiple prediction algoritms. MutationTaster2, Polyphen2, Provean, and SIFT were applied to predict the pathogenicity of missense SNVs (16-19). The pathogenic effect of in-frame indels was determined by FATHMM-Indel, Provean, SIFT-Indel, and VEST-Indel (18,20-22). Variants predicted as damaging by two or more algorithms were classified as pathogenic mutations. All the selected variants were then validated by Sanger sequencing using the ABI Prism BigDye Terminator v3.1 Cycle Sequencing Kit (Applied Biosystems) and results analysed as previously described (3). All primer used are listed in the Supplementary Table S3.

Determination of Whole Genome Copy Number Variations Whole-genome CNV was detected by CytoScanHD array platform (Affymetrix) according to manufacturer's protocol as previously described (3). Analysis was performed by Partek Genomics Suite 7.0 as previously described (1,3). Integrated results from individual patients and genomic regions recurrently and significantly amplified or deleted in our samples were identified by GISTIC2.0 (23).

PCR *IDH1* and *TERT promoter* *IDH1* was amplified from 20 ng genomic DNA with forward primer 5'- ACCAAATGGCACCATACGA-3' and reverse primer 5'-TTCATACCTTGCTTAATGGGTGT-3' using conditions as in (24). Two hotspot mutation, C228T and C250T in the *TERT* promoter were screened using the following primers: FW 5'-CAGACGCCCAGGACCGCGCT-3' and RV 5'-AGGGAGCGCACGGCTCGGCA-3'. PCR was performed using buffer condition of Taq Gold Polymerase DNA Polymerase (Thermo Fisher), in 25 ul reaction mixture containing 100ng of gDNA, with the addition of 5% DMSO and 2.5mM MgCl2. PCR was initiated at 94°C for 5 min, followed by 40 cycles of 94°C for 45s, 68°c for 45s, 72°c for 2 min whit a final extension of 72°c for 10min.

Analysis of *EGFRvIII* Status For the RT-PCR assay, total RNA was extracted from fresh-frozen tumor and GSCs samples using Direct-zol™ RNA MiniPrep Plus kit (Zymo Research, USA). Complementary DNA (cDNA) was synthesized used a SuperScript™ III Reverse Transcriptase (Thermo Fisher) following the manufactory protocol. The cDNA was amplified with the forward primer 5'-CTTCGGGGAGCAGCGATGCGAC-3' and reverse primer 5'-ACCAATACCTATTCCGTTACAC-3', designed specifically to amplify EGFR (1044bp product) and EGFRvIII (243bp product) respectively, according to (25). *Long range PCR amplification.* Genomic DNA was extracted from fresh-frozen tumor and GSCs samples with Blood & Cell Culture DNA Midi Kit (Qiagen) according to the manufactory's instructions. Long-range PCR was performed using TaKaRa LA Taq® DNA Polymerase (Takara) with 100 to 200 ng of genomic DNA. Genomic DNA was amplified using a forward mixture composed by 14 primers as describe in (26).

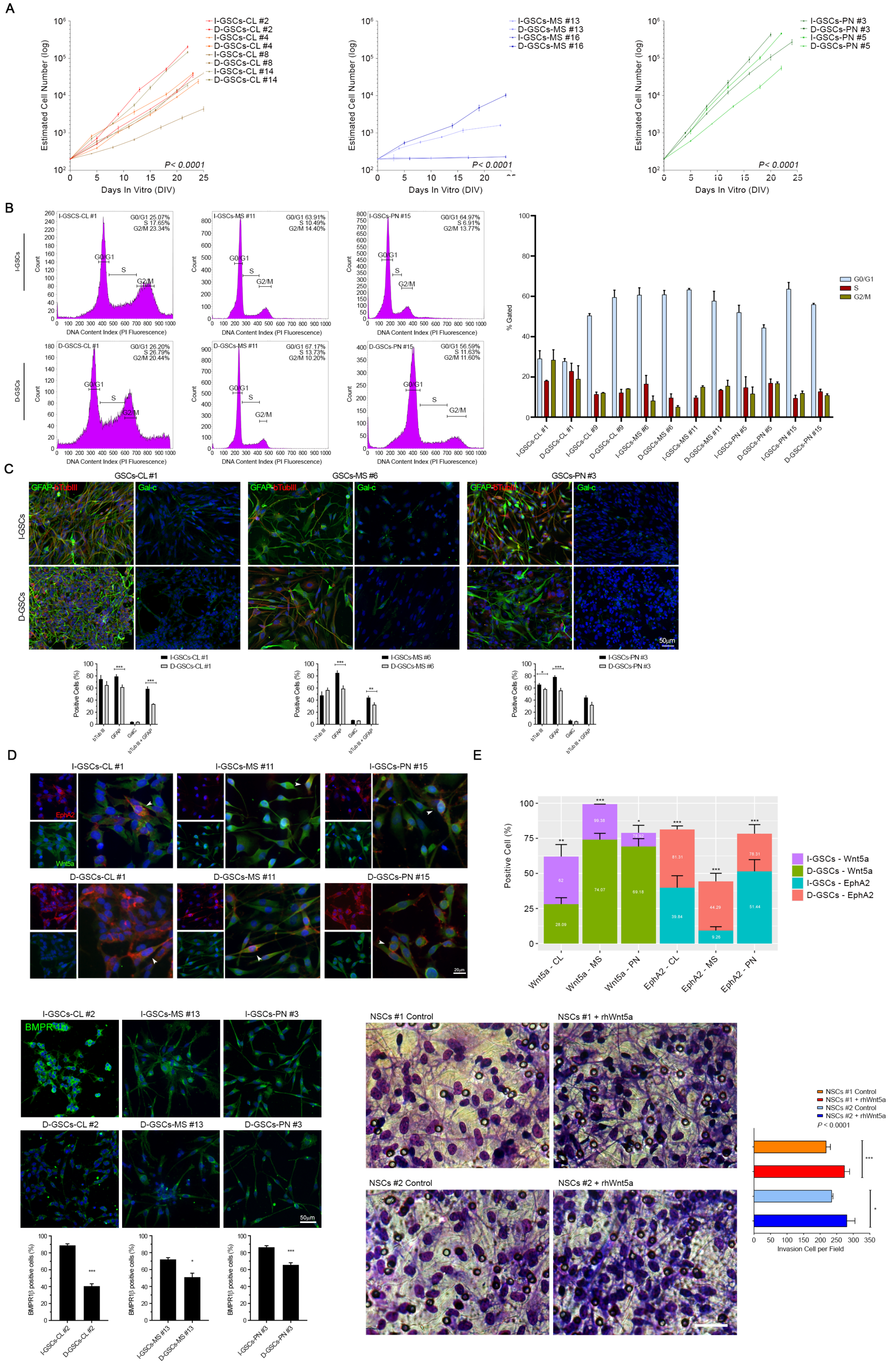
A
Estimated Cell Number (log)
Days In Vitro (DIV)
P< 0.0001
I-GSCs-CL #2
D-GSCs-CL #2
I-GSCs-CL #4
D-GSCs-CL #4
I-GSCs-CL #8
D-GSCs-CL #8
I-GSCs-CL #14
D-GSCs-CL #14
I-GSCs-MS #13
D-GSCs-MS #13
I-GSCs-MS #16
D-GSCs-MS #16
I-GSCs-PN #3
D-GSCs-PN #3
I-GSCs-PN #5
D-GSCs-PN #5
B
I-GSCs
D-GSCs
Count
DNA Content Index (PI Fluorescence)
I-GSCS-CL #1
G0/G1 25.07%
S 17.65%
G2/M 23.34%
I-GSCs-MS #11
G0/G1 63.91%
S 10.49%
G2/M 14.40%
I-GSCs-PN #15
G0/G1 64.97%
S 6.91%
G2/M 13.77%
D-GSCs-CL #1
G0/G1 26.20%
S 26.79%
G2/M 20.44%
D-GSCs-MS #11
G0/G1 67.17%
S 13.73%
G2/M 10.20%
D-GSCs-PN #15
G0/G1 56.59%
S 11.63%
G2/M 11.60%
% Gated
G0/G1
S
G2/M
C
GSCs-CL #1
GSCs-MS #6
GSCs-PN #3
GFAP-bTubIII
Gal-c
50μm
Positive Cells (%)
I-GSCs-CL #1
D-GSCs-CL #1
I-GSCs-MS #6
D-GSCs-MS #6
I-GSCs-PN #3
D-GSCs-PN #3
D
I-GSCs-CL #1
I-GSCs-MS #11
I-GSCs-PN #15
D-GSCs-CL #1
D-GSCs-MS #11
D-GSCs-PN #15
EphA2
Wnt5a
20μm
E
Positive Cell (%)
I-GSCs - Wnt5a
D-GSCs - Wnt5a
I-GSCs - EphA2
D-GSCs - EphA2
Wnt5a - CL
Wnt5a - MS
Wnt5a - PN
EphA2 - CL
EphA2 - MS
EphA2 - PN
I-GSCs-CL #2
I-GSCs-MS #13
I-GSCs-PN #3
BMPR-1b
D-GSCs-CL #2
D-GSCs-MS #13
D-GSCs-PN #3
50μm
BMPR1β positive cells (%)
NSCs #1 Control
NSCs #1 + rhWnt5a
NSCs #2 Control
NSCs #2 + rhWnt5a
P < 0.0001
Invasion Cell per Field

Supplementary Figure 1. ***Ex vivo*** **functional profile of I-GSCs cells. A.** Growth kinetic of I-GSCs lines is significantly lower as compared to that one of their cognate D-GSCs cells, regardless of subtype. ***P<0.001, I-GSCs vs. D-GSCs, hierarchical linear model for repeated measurements. **B.** Analysis of cell cycle progression of I- and D-GSCs across subtype showing the former with a higher proportion of cells in G0/G1 phase, whereas the latter in S phase. **C.** Immunofluorescence representative images reporting the multipotential capacity of I- (top) and D-GSCs (bottom) across subtype, the former overexpressing astrocytic-like cells (GFAP; green) as compared to the latter. The level of neuronal- and oligodendrocyte-like cells (class III b-tubulin and GalC; red and green, respectively) is shown to be unchanged (left). Several co-localization of proteins are denoted (yellow). *Bar*, 50um. ***P<0.001, ** P<0.01 *P<0.05, one-way Student's *t*-test. **D.** Confocal images depicting a widespread and intense immunoreactivity for Wnt5a protein (green) and a less obvious and intense signal for EphA2 (red) in I-GSCs cells across molecular subclusters (top, left). By contrast, D-GSCs cells reported a strong positivity for EphA2 and a weak and scanty signal for Wnt5a (bottom, left). Infrequent co-expression of Wnt5a and EphA2 in both GSCs populations (yellow) is arrowhead. *Bar*, 20um. Quantification of both markers is shown (right). ***P<0.001, **P<0.01, *P<0.05, unpaired Student's *t*-test. **E.** By means of confocal imaging, I-GSCs display a higher percentage of BMPR1b positive cells as compared to their sibling D-GSCs. *Bar*, 50um. ***P<0.001, *P<0.05, one-way Student's *t*-test. **F.** *In vitro* migration assays reporting that enhancing Wnt5a expression in NSCs cells by exposure to rhWnt5a improve their rate of migration. *Bar*, 50um. ***P<0.001, *P<0.05, one-way Student's *t*-test.

Quantification in **A** is shown as mean ± SD, in **B-F** as mean ± SEM from at least three independent experiments.

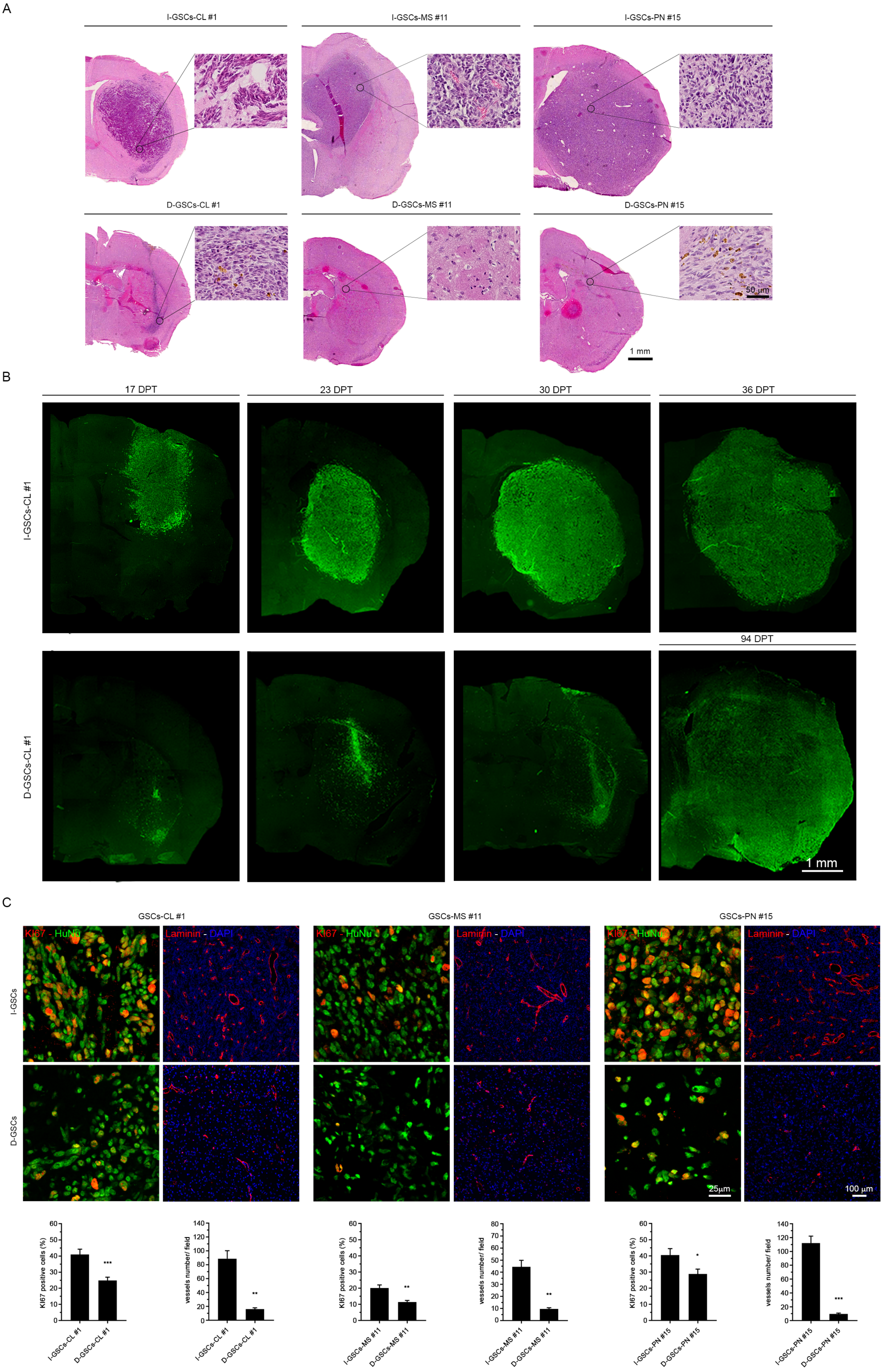
A
I-GSCs-CL #1
I-GSCs-MS #11
I-GSCs-PN #15
D-GSCs-CL #1
D-GSCs-MS #11
D-GSCs-PN #15
50 µm
1 mm
B
17 DPT
23 DPT
30 DPT
36 DPT
94 DPT
I-GSCs-CL #1
D-GSCs-CL #1
1 mm
C
GSCs-CL #1
GSCs-MS #11
GSCs-PN #15
KI67 - HuNu
Laminin - DAPI
I-GSCs
D-GSCs
25µm
100 µm
KI67 positive cells (%)
vessels number/ field
I-GSCs-CL #1
D-GSCs-CL #1
I-GSCs-MS #11
D-GSCs-MS #11
I-GSCs-PN #15
D-GSCs-PN #15

Supplementary Figure 2. The highly *in vivo* tumorigenic profile of I-GSCs cells. A. Representative haematoxylin & eosin-stained sections illustrating, irrespectively of the subtype, increased tumor development and infiltration and intraparenchymal edema possible cojoined to tumor-related inflammatory response (arrowhead) in I-GSCs-injected mice as compared to their D-GSCs counterpart (*n*=5 mice). Insets: higher magnification reporting the distinctive histological characteristics of GBMs, such as nuclear atypia, mitoses, edema, neo-angiogenesis and microvascular proliferation. *Bars*, 50 um and 1 mm. **B.** Representative serial histological reconstruction of mouse brain sections from experiment in A showing that luciferase-tagged I-GSCs establish intracranial GBM with a strikingly faster aggressive patterns than those of D-GSCs cells. *Bar*, 1mm. **C.** Confocal representative images depicting increasing mitotic activity and vascular proliferation in I-GSCs-derived tumors mice versus their D-GSCs sibling, with a more intense immunoreactivity for KI67 (red; left panels) and a widespread laminin (red; right panels) positivity. Yellow cells mark the co-localization of KI67 with the human-specific marker HuNu. *Bars*, 25um and 100um. Quantification is shown as mean ± SEM (bottom panels). ***P<0.001, **P<0.01, *P<0.05 one-way Student's *t*-test.

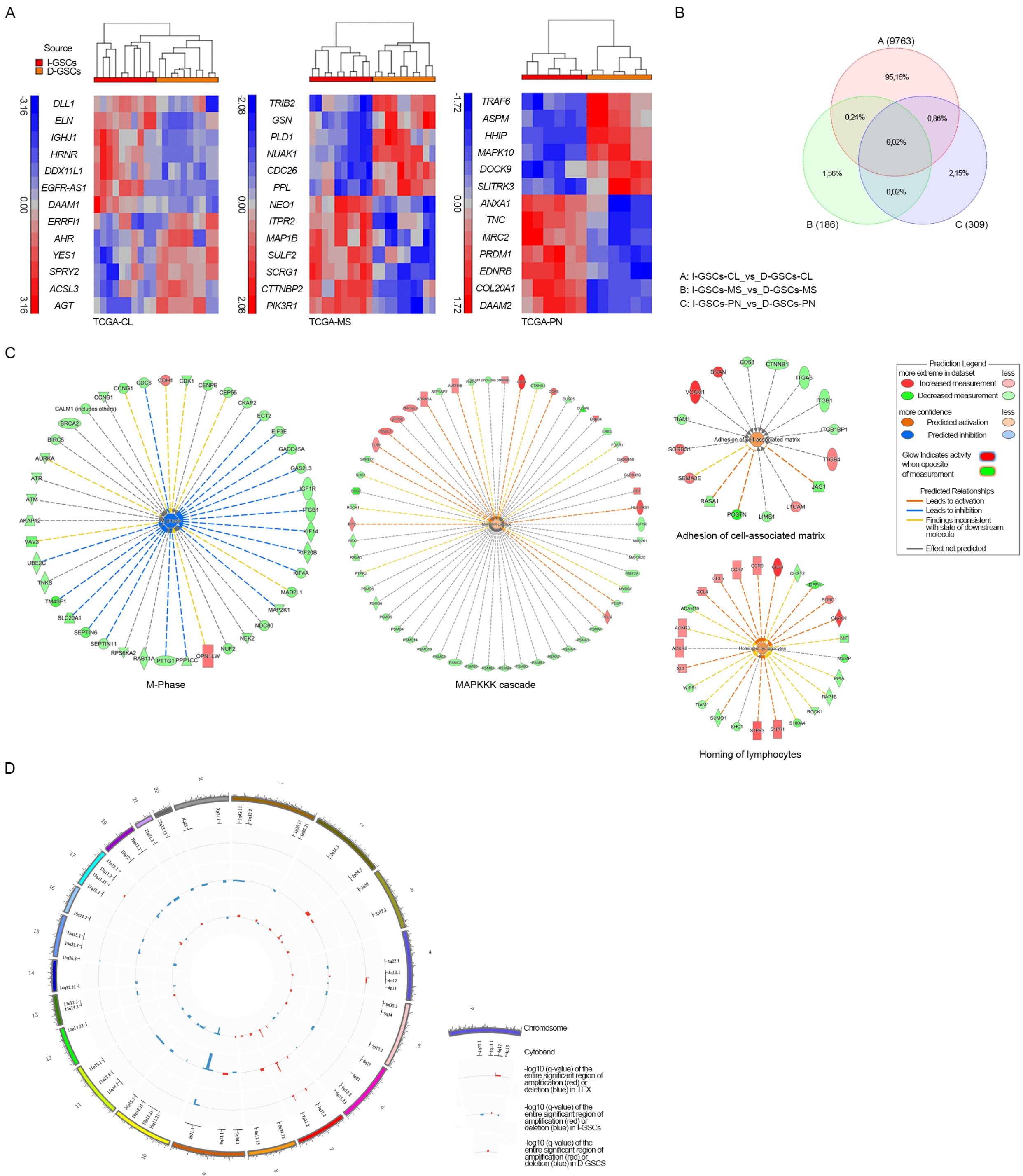

Supplementary Figure 3. A migratory molecular signature is distinctive for I-GSCs as compared to their matched GSCs lines. A. Unsupervised hierarchical clustering analysis based on gene expression of selected genes illustrating the enrichment of genes involved in degradation of cellular matrix, cell movement and differentiation processes in I-GSCs cells versus their D-GSCs siblings across subtypes. A dual color code represents up- (red) and downregulated (blue) genes. **B.**

The number of genes differentially expressed in classical I-GSCs as compared to their sibling D-GSCs is higher than one emerged from the comparison between mesenchymal and proneural GSCs, as depicted by Venn diagrams. **C.** Ingenuity associated functional analysis confirming the enrichment of genes involved in cell cycling and proliferation in D-GSCs, while genes controlling extracellular communication and a pro-inflammatory state are peculiar of I-GSCs. **D.** Circos plot reporting gene copy number change profiling of both GSCs subsets and of their tissue of origin and the cytobands involved, as detected by GISTIC. -log10(*q*-value) of the significant region of amplification or deletion is in red or blue, respectively.

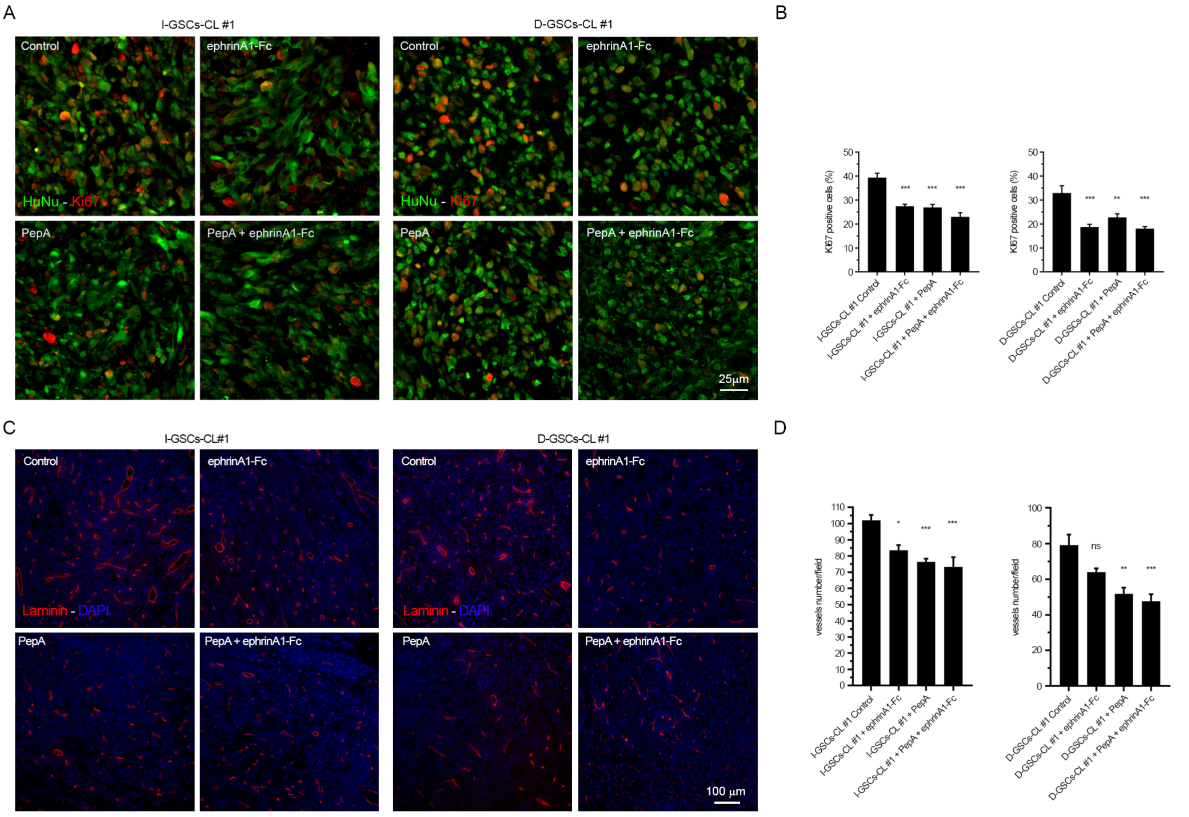

Supplementary Figure 4. Simultaneous infusion of PepA and ephrinA1-Fc additively hinders GSCs tumorigenicity. A-B. Confocal representative images reporting that the combination of PepA and ephrinA1-Fc is superior to the single treatments alone in terms of significantly depleting the percentage of mitotically active GSCs (KI67) in tumors established from either I- and D-GSCs cells. *Bar*, 25um (**A**). Quantification is shown in **B** as mean ± SEM. ****P*<0.001, ***P*<0.01, ANOVA Tukey's multiple comparison test. **C-D.** PepA infusion in combination with ephrinA1-Fc display significant additive effect in lessening the number of vessels (Laminin) in GBM tumors. *Bar*, 100um (**C**). Quantification is shown in **D** as mean ± SEM. ****P*<0.001, ***P*<0.01, ns not significant, ANOVA Tukey's multiple comparison test.

References

1. Binda E, Visioli A, Giani F, Trivieri N, Palumbo O, Restelli S*, et al.* Wnt5a Drives an Invasive Phenotype in Human Glioblastoma Stem-like Cells. Cancer Res **2017**;77:996-1007
2. Galli R, Binda E, Orfanelli U, Cipelletti B, Gritti A, De Vitis S*, et al.* Isolation and characterization of tumorigenic, stem-like neural precursors from human glioblastoma. Cancer Res **2004**;64:7011-21
3. Visioli A, Giani F, Trivieri N, Pracella R, Miccinilli E, Cariglia MG*, et al.* Stemness underpinning all steps of human colorectal cancer defines the core of effective therapeutic strategies. EBioMedicine **2019**;44:346-60
4. Trivieri N, Pracella R, Cariglia MG, Panebianco C, Parrella P, Visioli A*, et al.* BRAF V^{600E} mutation impinges on gut microbial markers defining novel biomarkers for serrated colorectal cancer effective therapies. J Exp Clin Cancer Res **2020**;39:285
5. Jenei V, Sherwood V, Howlin J, Linnskog R, Säfholm A, Axelsson L*, et al.* A t-butyloxycarbonyl-modified Wnt5a-derived hexapeptide functions as a potent antagonist of Wnt5a-dependent melanoma cell invasion. Proc Natl Acad Sci U S A **2009**;106:19473-8
6. Davis FG, Freels S, Grutsch J, Barlas S, Brem S. Survival rates in patients with primary malignant brain tumors stratified by patient age and tumor histological type: an analysis based on Surveillance, Epidemiology, and End Results (SEER) data, 1973-1991. J Neurosurg **1998**;88:1-10
7. Liu DP, Wang Y, Koeffler HP, Xie D. Ephrin-A1 is a negative regulator in glioma through down-regulation of EphA2 and FAK. Int J Oncol **2007**;30:865-71
8. Binda E, Visioli A, Giani F, Lamorte G, Copetti M, Pitter KL*, et al.* The EphA2 receptor drives self-renewal and tumorigenicity in stem-like tumor-propagating cells from human glioblastomas. Cancer Cell **2012**;22:765-80

9. Wood LD, Parsons DW, Jones S, Lin J, Sjoblom T, Leary RJ, *et al.* The genomic landscapes of human breast and colorectal cancers. Science **2007**;318:1108-13

10. Verhaak RG, Hoadley KA, Purdom E, Wang V, Qi Y, Wilkerson MD, *et al.* Integrated genomic analysis identifies clinically relevant subtypes of glioblastoma characterized by abnormalities in PDGFRA, IDH1, EGFR, and NF1. Cancer Cell **2010**;17:98-110

11. Network CGAR. Comprehensive genomic characterization defines human glioblastoma genes and core pathways. Nature **2008**;455:1061-8

12. Li H, Durbin R. Fast and accurate short read alignment with Burrows-Wheeler transform. Bioinformatics **2009**;25:1754-60

13. DePristo MA, Banks E, Poplin R, Garimella KV, Maguire JR, Hartl C, *et al.* A framework for variation discovery and genotyping using next-generation DNA sequencing data. Nat Genet **2011**;43:491-8

14. Koboldt DC, Zhang Q, Larson DE, Shen D, McLellan MD, Lin L, *et al.* VarScan 2: somatic mutation and copy number alteration discovery in cancer by exome sequencing. Genome Res **2012**;22:568-76

15. Wang K, Li M, Hakonarson H. ANNOVAR: functional annotation of genetic variants from high-throughput sequencing data. Nucleic Acids Res **2010**;38:e164

16. Schwarz JM, Cooper DN, Schuelke M, Seelow D. MutationTaster2: mutation prediction for the deep-sequencing age. Nat Methods **2014**;11:361-2

17. Adzhubei I, Jordan DM, Sunyaev SR. Predicting functional effect of human missense mutations using PolyPhen-2. Curr Protoc Hum Genet **2013**;Chapter 7:Unit7.20

18. Choi Y, Chan AP. PROVEAN web server: a tool to predict the functional effect of amino acid substitutions and indels. Bioinformatics **2015**;31:2745-7

19. Sim NL, Kumar P, Hu J, Henikoff S, Schneider G, Ng PC. SIFT web server: predicting effects of amino acid substitutions on proteins. Nucleic Acids Res **2012**;40:W452-7

20. Ferlaino M, Rogers MF, Shihab HA, Mort M, Cooper DN, Gaunt TR*, et al.* An integrative approach to predicting the functional effects of small indels in non-coding regions of the human genome. BMC Bioinformatics **2017**;18:442

21. Hu J, Ng PC. SIFT Indel: predictions for the functional effects of amino acid insertions/deletions in proteins. PLoS One **2013**;8:e77940

22. Douville C, Masica DL, Stenson PD, Cooper DN, Gygax DM, Kim R*, et al.* Assessing the Pathogenicity of Insertion and Deletion Variants with the Variant Effect Scoring Tool (VEST-Indel). Hum Mutat **2016**;37:28-35

23. Mermel CH, Schumacher SE, Hill B, Meyerson ML, Beroukhim R, Getz G. GISTIC2.0 facilitates sensitive and confident localization of the targets of focal somatic copy-number alteration in human cancers. Genome Biol **2011**;12:R41

24. Balss J, Meyer J, Mueller W, Korshunov A, Hartmann C, von Deimling A. Analysis of the IDH1 codon 132 mutation in brain tumors. Acta Neuropathol **2008**;116:597-602

25. Mellinghoff IK, Wang MY, Vivanco I, Haas-Kogan DA, Zhu S, Dia EQ*, et al.* Molecular determinants of the response of glioblastomas to EGFR kinase inhibitors. N Engl J Med **2005**;353:2012-24

26. Frederick L, Eley G, Wang XY, James CD. Analysis of genomic rearrangements associated with EGRFvIII expression suggests involvement of Alu repeat elements. Neuro Oncol **2000**;2:159-63
