# Supplementary material for: Growth factor independence underpins a paroxysmal, aggressive Wnt5aHigh/EphA2Low phenotype in glioblastoma stem cells, conducive to experimental combinatorial therapy
Source: J Exp Clin Cancer Res. 2022 Apr 12;41:139. doi: 10.1186/s13046-022-02333-1 (PMC9004109; doi:10.1186/s13046-022-02333-1)
Supplement: Supplementary file 2 — Additional file 2. [file 13046_2022_2333_MOESM2_ESM.docx]

**Table S1.** Ingenuity Analysis of the I- and D-GSCs Genes Demonstrating the Associated Cancer Genes Molecular and Molecular and Cellular Functions.

| Categories | Functions | Diseases or Functions Annotation | # Molecules | p-value | Molecules |
| --- | --- | --- | --- | --- | --- |
| Cell Death and Survival | survival | Cell survival | 241 | 1,10E-06 | ACKR3,ACTL6A,ADM,AIMP1,AK5,ANTXR2,ARAP3,ARRB1,ATF3,ATM,ATP5MD,ATR,AURKA,B4GALT5,BCLAF1,BEX3,BIRC5,BMPR1A,BNIP2,BNIP3,BRCA2,CALB1,CAMK2D,CAPN3,CASP3,CASP7,CCL4,CCL5,CCNA2,CCNB1,CCNG1,CCNG2,CCR7,CD151,CD38,CD74,CD82,CDC40,CDH1,CDH5,CDK1,CDK6,CHKA,CLOCK,COPS4,COPS5,CRYAB,CTNNB1,CX3CR1,DCK,DCUN1D5,DGKB,DIS3,DPP4,DUSP5,DUSP6,EBNA1BP2,ECT2,EIF2S1,EIF3E,EMP1,ENO1,EPAS1,EPHA2,EPHB1,EPHB2,ERBB4,EREG,ERRFI1,ESCO1,ETV5,FANCM,FBL,FGFR1,FH,FKBP5,FUS,GADD45A,GADD45B,GLS,GPNMB,GPR17,GPR180,GRB10,GTPBP4,GUCY1A1,HAPLN1,HEY1,HGF,HIF1A,HK1,HK2,HMGA2,HNRNPUL2,HSPB8,ID2,IGF1R,IGF2BP3,IGFBP2,IKZF2,INSIG1,ITGA6,ITGB1,ITGB1BP1,JAG1,JUN,KIF18A,L1CAM,LDHA,LGALS3,LIMS1,LSM6,LSM8,MAD2L1,MALT1,MAP2K1,MAP3K19,MAP3K8,MAPK10,MAPT,MCUR1,MGAT5,MIF,MIS18A,MSH2,MSH6,MUC20,MX1,NAB1,NCK1,NDC80,NDRG1,NDUFAF4,NEAT1,NLK,NOS2,NR4A2,NR4A3,NRN1,NUAK1,NUF2,NUP54,PBK,PDHA1,PDK1,PDRG1,PFDN4,PFKFB2,PIK3CA,PLK4,PLP1,POMP,POSTN,PPAT,PPIA,PPM1B,PPP1CC,PPP2R1B,PPP2R5C,PPP3CA,PREP,PRKAR1A,PRMT1,PRPF3,PSMA1,PSMA3,PSMA4,PSMA7,PSMB4,PSMB5,PSMB6,PSMC5,PSMD14,PTPRG,PTTG1,RAB11A,RAD51C,RAD54B,RALB,RAMAC,RASA1,RASSF8,RBM39,RFPL4A/RFPL4AL1,RHOJ,RPF1,RPL24,RPL35A,RPS6KA2,RPS6KA3,RPSA,RRM1,RRM2B,S100A4,S1PR1,S1PR3,SELENOS,SERPINA3,SERPINI1,SH3KBP1,SHC1,SLPI,SNAI2,SNCAIP,SPATS2,SPN,SPRY1,SUGT1,SUMO1,TAF1B,TBK1,TIMP1,TLR3,TLR9,TNFRSF10B,TNKS,TOPBP1,TOX,TPT1,TRAF6,TRIB2,TSG101,TTK,TXNL4A,TYMS,UCHL5,UGCG,USP13,USP38,USP47,VCAM1,VHL,VRK1,WIPF1,YAP1,YEATS4,YES1,ZEB1,ZFP36,ZNF429,ZNF43 |
| Cellular Development, Cellular Growth and Proliferation | cell proliferation | Cell proliferation of tumor cell lines | 255 | 1,74E-03 | AASDHPPT,ACAT1,ACER3,ACKR3,ACSL4,ADAM10,ADM,AHSA1,AKAP12,AMOTL2,ARHGAP11A,ARL5A,ATF3,ATM,ATP5MD,ATR,AURKA,B3GAT1,BIRC5,BMPR1A,BMPR1B,BNIP3,BRCA2,BRK1,CAMK2D,CAPN3,CASP3,CASP7,CCDC80,CCNA2,CCNB1,CCNB2,CCNG2,CD151,CD38,CD74,CDC16,CDCA2,CDH1,CDK1,CDK6,CDKN3,CELF2,CEMIP2,CENPE,CHKA,CIP2A,CIRBP,CISD1,CMC2,CMTM5,COL4A2,COPS4,COPS5,COPS6,CRYAB,CTNNB1,CTR9,CUL5,DACH1,DCLK1,DEPDC1,DLGAP5,DLL1,DPP4,DUSP4,DUSP5,DUSP6,ECT2,EIF3E,EIF4H,EIF5A2,EMP1,EPAS1,EPHA2,ERBB4,EREG,ERRFI1,ETV4,EXTL3,FDFT1,FEN1,FGFR1,FH,FHL1,FKBP5,FOXF2,FRMD6,FUS,FUT8,FZD7,GADD45A,GADD45G,GLS,GMNN,GNG7,GNL3,GRK5,GTPBP4,H1-3,H2AZ1,HGF,HIF1A,HIF1A-AS2,HK1,HK2,HMGA2,HNRNPA1,HNRNPA2B1,HSPB8,ID2,IFT88,IGF1R,IGF2BP3,IGFBP2,IKZF2,IMMT,INPP5F,IPO7,ITGA6,ITGB1,ITGB4,JAG1,JUN,KCNK2,KIF2C,KIR3DL1,L1CAM,LAMTOR5,LDHA,LGALS1,LGALS3,LUZP2,MAD2L1,MAF,MALT1,MAP2K1,MBD2,MDGA2,MGAT5,MIF,MNAT1,MSI2,MSTN,MUC1,MUC16,MYBL1,NCAPG,NDRG1,NDUFAF4,NEAT1,NEK2,NELFCD,NGEF,NMUR2,NOS2,NR4A2,NUCB2,OAZ1,PBK,PDS5B,PEBP1,PFKP,PHLDA1,PIK3CA,PLAG1,PLD1,POSTN,PPP1CC,PPP2R5C,PRKAR1A,PRMT1,PRPF3,PRPF4B,PSMA4,PSMB3,PSMD4,PTPRG,PTPRJ,PTTG1,RALB,RAP1B,RASSF5,RASSF8,RBX1,RFC4,RFPL4A/RFPL4AL1,RHOJ,RPS25,RPS6KA3,RPSA,RRM1,S100A4,S1PR3,SFRP4,SH2B3,SHC1,SHCBP1,SLC20A1,SLC26A2,SLC36A4,SLC38A7,SLC7A11,SLC9A3R1,SLFN5,SLPI,SNAI2,SOCS6,SPRY2,SPRY4,SRP54,STOML2,STX6,SUMO1,TEAD1,TIAM1,TIAM2,TIFA,TIPRL,TLR3,TMPO,TMSB10/TMSB4X,TNFRSF10B,TNKS,TOPBP1,TOX,TPT1,TRAF6,TRIB2,TSG101,TYMS,UAP1,UBA2,UBA6,UBE2C,UBE2J1,UBE2M,UCA1,UGCG,URI1,USP12,USP17L2 (includes others),UXT,VAV3,VCAM1,VDAC3,VHL,YAP1,YEATS4,YES1,ZBED6,ZEB1,ZFP36,ZNF143,ZNF404 |
| Cancer, Organismal Injury and Abnormalities | growth | Growth of tumor | 142 | 3,49E-03 | ACAT1,ACKR3,ACSL4,ACSS2,ADAM10,ADGRG1,ADM,AIMP1,ATF3,ATM,AURKA,BACH1,BCAN,BIRC5,BNIP3,BNIP3L,C3AR1,CA12,CASP3,CCL5,CD151,CD34,CD38,CD82,CDC6,CDH1,CDH5,CDK1,CDK6,CIP2A,CISD1,COL4A1,COX17,CTNNB1,CTNND2,CX3CR1,DACH1,DLL1,DPP4,EPAS1,EPHA2,EPHB2,ERO1A,FGFR1,FH,FHL1,FOXF2,FOXJ1,FRMD6,FUT8,FZD7,GADD45A,GALNT1,GLS,GPNMB,HGF,HIF1A,HK2,HMGA2,ID2,IFT88,IGF1R,IGFBP2,IKZF2,ITGB1,JAG1,JUN,L1CAM,LAMTOR5,LDHA,LGALS1,LGALS3,LRRC4,MALT1,MAP2K1,MAP3K8,MGAT5,MIF,MSI2,MSTN,MUC1,MUC20,NCAPG,NDRG1,NOS2,NR4A2,NUAK1,PDHA1,PDK1,PGGT1B,PHLDA1,PIK3CA,PLD1,POSTN,PREP,PRKAR1A,PSMB4,RALB,RASA1,RASSF8,RPS19,RPS4X,S1PR3,SEMA7A,SH2B3,SHC1,SHCBP1,SLC20A1,SLC4A7,SLC7A11,SLPI,SNAI2,SPN,SPRY4,SUGT1,TBK1,TEAD1,TIAM1,TIAM2,TIGAR,TIMP1,TLR3,TLR9,TNFRSF10B,TNKS,TOX,TPT1,TRIB2,UBA2,UBE2C,URI1,USP12,VAV3,VHL,YAP1,YEATS4,YES1,ZEB1,ZFP36,ZMYND10,ZNF143,ZNF326 |
| Cell Cycle | mitosis | Mitosis | 83 | 2,89E-04 | ACTL6A,ADM,ARPC1B,ARRB1,ATM,ATR,AURKA,BIRC5,BMPR1B,BORA,BRCA2,CCNA2,CCNB1,CCNB1IP1,CCNB2,CDC6,CDK1,CDKN3,CENPE,CENPW,CHMP2A,CHMP4B,CKAP2,CLASP1,DCLK1,DLGAP5,DYNLT3,EIF3E,ERBB4,EREG,ERRFI1,FGFR1,GADD45A,GADD45B,GADD45G,GAS1,GMNN,GNL3,HAUS4,HGF,IGF1R,IGFBP2,JUN,KIF18A,KIF2C,KIF4A,LAMTOR5,MAD2L1,MAP2K1,MIF,MIS18A,NAE1,NDC80,NEK2,NUF2,PDS5B,PEBP1,PLK4,PTTG1,RFPL4A/RFPL4AL1,RPL24,RPS6,RPS6KA3,S1PR1,SHC1,SHROOM2,SLC20A1,SLC9A3R1,SOCS5,SPAG5,SPRY2,SUGT1,TLR9,TNKS,TSG101,TTK,UBE2C,UCHL5,USP16,VAV3,WWC1,YAP1,ZFP36 |
| Cell Cycle, Cellular Movement | cytokinesis | Cytokinesis | 30 | 1,27E-03 | AURKA,BIRC5,BRCA2,CALM1 (includes others),CCNB1,CCNG1,CDC6,CDH1,CEP55,CKAP2,ECT2,EIF3E,GADD45A,GAS2L3,IGF1R,ITGB1,KIF14,KIF20B,KIF4A,MAP2K1,NEK2,OPN1LW,PPP1CC,RAB11A,SEPTIN11,SEPTIN6,SLC20A1,TM4SF1,UBE2C,VAV3 |
| Cell Cycle | mitotic index | Mitotic index | 8 | 2,69E-03 | AURKA,BIRC5,GADD45A,IGFBP2,MAD2L1,MIF,TSG101,TTK |
| Protein Synthesis | metabolism | Metabolism of protein | 171 | 6,72E-04 | ACO1,ADAM10,ADAM19,ADAMTS9,ADI1,ADM,AFG3L2,ANAPC10,ANAPC16,APLP2,ARRB1,ATM,ATP1B3,AURKA,B4GALT5,CALB1,CAPN3,CASP3,CASP7,CCKAR,CCT8,CD46,CDC16,CDH1,CENPE,CHCHD1,CIRBP,CMTM6,CNBP,COPS5,CREBL2,CRYAB,CTNNB1,CTSC,CYP51A1,DARS1,DHPS,DLD,DPP4,EDEM1,EDEM3,EIF2S1,EIF3E,EIF3K,EIF4A2,EIF4H,ERRFI1,FAM107A,FBXW2,FUCA2,FUS,GAL3ST4,GTPBP4,H1-5,H2AZ1,H3C1,H3C10,H3C3,H3C8,H4-16,HNRNPD,HSPB8,IFIT1,IGF2BP2,IGF2BP3,IGFBP2,IMPACT,IREB2,ITGB1,JUN,KLHL15,KLHL8,KRR1,KTN1,LAMC1,LDLR,LGALS1,LPL,MAD2L1,MALT1,MAP2K1,MAP3K8,MAPT,MEF2A,MGAT4A,MIB1,MID1,MMP15,MRPL13,MRPL16,MRPL17,MRPL19,MRPL20,MRPL22,MRPL32,MRPL33,MRPL34,MRPL39,MRPL44,MRPL47,MRPL51,MRPL53,MRPL58,MRPS15,MRPS23,MRPS28,MRPS6,MSTN,NAT8B,NCBP2,NCK1,NDC80,NLK,NOS2,NPC1L1,OAZ1,OAZ2,PARL,PCYOX1,PGA5 (includes others),PIK3CA,PLD1,PM20D2,PPAT,PREP,PSMB3,PSMB5,PSMD14,PTTG1,RASA1,RBM3,RNF168,RPL17,RPL23,RPL24,RPL27A,RPL35A,RPL36,RPL5,RPS15A,RPS16,RPS19,RPS25,RPS3A,RPS4X,RPS5,RPS6,RPS7,RPSA,RRBP1,S100A4,SCG2,SELENOS,SNCAIP,SPCS3,STT3B,SUGT1,SUMO1,SWAP70,TIMP1,TMPRSS7,TSG101,TSPAN5,TYMS,UBA6,UBE2C,UBE2K,USP13,UTP18,VHL,ZFP36 |
| Cell Cycle | M phase | M phase | 41 | 2,63E-04 | AKAP12,ATM,ATR,AURKA,BIRC5,BRCA2,CALM1 (includes others),CCNB1,CCNG1,CDC6,CDH1,CDK1,CENPE,CEP55,CKAP2,ECT2,EIF3E,GADD45A,GAS2L3,IGF1R,ITGB1,KIF14,KIF20B,KIF4A,MAD2L1,MAP2K1,NDC80,NEK2,NUF2,OPN1LW,PPP1CC,PTTG1,RAB11A,RPS6KA2,SEPTIN11,SEPTIN6,SLC20A1,TM4SF1,TNKS,UBE2C,VAV3 |
| Cell Cycle | G1/S phase transition | G1/S phase transition | 43 | 1,01E-04 | ADRA1A,ATF3,BCAT1,BIRC5,CAMK2D,CCNA2,CCNG1,CDC6,CDK1,CDK6,CDKN3,COPS5,CTNNB1,CUL5,DARS1,GADD45A,GADD45B,GADD45G,GMNN,GNL3,GPI,HGF,HIF1A,ID2,IGF1R,ITGB1,JUN,MAP2K1,MCM6,MDGA2,MNAT1,MX2,NUAK1,PLAG1,POLE2,PPP3CA,RPL23,RPL5,RPS6,SPRY2,TOPBP1,TSG101,UCA1 |
| Metabolic Disease, Organismal Injury and Abnormalities | glucose metabolism disorder | Glucose metabolism disorder | 208 | 1,65E-03 | ABCC9,ACAT2,ACO1,ADAMTS9,ADM,ADRA1A,ANAPC10,APOO,ARL1,ATF3,ATM,ATP1B3,ATP5F1E,ATP5MPL,BMPR1A,BRCA2,BTG1,C1orf162,C3AR1,CA12,CA13,CACNB2,CALB1,CAPN3,CASP3,CCDC25,CCL3L3,CCL4,CCL5,CCNB1IP1,CCNG1,CCR7,CCR9,CD34,CD38,CD5L,CD74,CDC42EP3,CDK6,CHST7,CIITA,CISD1,CLDN16,CMKLR1,COL4A1,COL4A2,COPS5,COX6C,COX7B,COX7C,CRIM1,CRY1,CTNNB1,CX3CR1,CYP51A1,DAAM1,DECR1,DGKB,DHRS3,DPP4,EDNRB,ELMO1,EPAS1,EXOSC9,FCGR1B,FDFT1,FGFR1,FKBP5,FRMD4B,GANAB,GAS1,GBE1,GBP4,GDPD2,GFAP,GGCT,GLIS3,GPNMB,GPR1,GRB10,GRB14,GSS,GUCY1A1,HAPLN1,HERC6,HGF,HK2,HLA-DMA,HLA-DMB,HLA-DOA,HLA-DPA1,HLA-DPB1,HLA-DQA1,HLA-DQA2,HLA-DRA,HLA-DRB5,HTR2A,HTR3E,IER3IP1,IFIT1,IFNGR2,IGF1R,IGF2BP2,IGFBP2,IMMT,JUN,KBTBD2,KCNA4,KIF20B,L1CAM,LAMTOR5,LDLR,LGALS3,LPL,LRP8,LY6G5C,LY6G6C,MAPK10,MC3R,MGAM,MGAT2,MGAT4A,MGAT5,MGP,MIF,MRPL47,MRPL53,MSTN,MUC21,MUCL3,NAA25,NCF1,NDUFA4,NELFCD,NOS2,NPC1L1,NR4A2,NR4A3,NUCKS1,PARD3B,PDE7B,PDK1,PDYN,PHTF1,PIEZO2,PKIB,POLI,PPIA,PPP1R3B,PPP3CA,PPP3R1,PRDX3,PREP,PREX2,PRMT1,PSMA1,PSMA3,PSMB1,PSMB5,PSMC6,PSMD14,PSMD6,PSMD8,PTTG1,RAPGEF5,ROCK1,RPL17,RPL23,RPS15A,RPS3A,RPS6KA3,RPS7,RSAD2,RTP4,S100A4,SERPINA3,SH2B3,SIRPG,SLC44A5,SLC7A11,SLC9A9,SPN,SPOCK1,SPRED1,STRN3,STXBP3,SYNJ2,TFAM,TIMP1,TLR3,TLR9,TMC7,TPT1,TRPV5,TSPAN18,TSPAN5,TWSG1,UBE2K,UBE2M,USP12,USP16,VCAM1,VEPH1,VHL,XCL1,YBX3,ZBTB1,ZMAT4 |
| Cellular Movement | invasion | Invasion of kidney cancer cell lines | 7 | 1,44E-03 | FH,HGF,HIF1A,MUC1,TIAM1,TIMP1,VHL |
| Cell-mediated Immune Response, Cellular Movement, Hematological System Development and Function, Immune Cell Trafficking | trafficking | Trafficking of T lymphocytes | 8 | 6,06E-03 | CCL3L3,CCL4,CCL5,CCR7,CCR9,RAP1B,S1PR1,SPN |
| Inflammatory Disease, Neurological Disease | inflammatory demyelinating disease | Inflammatory demyelinating disease | 38 | 6,84E-03 | ADAM10,ADRA1A,AK5,AQP4,ARRB1,BCHE,CRYAB,CYP51A1,DCK,DHCR7,ERVW-1,FCGR1B,FDFT1,HLA-DMA,HLA-DMB,HLA-DQA1,HLA-DQA2,HLA-DRA,HLA-DRB1,HNRNPA1,IFIT1,ISG15,KCNA4,MAP2K1,MAPT,MIF,MX1,NDUFS4,NOS2,PLP1,POLE2,RPL5,RPS6,RRM1,RRM2B,RSAD2,S1PR1,TIMP1 |
| Cell Death and Survival | apoptosis | Apoptosis of tumor cell lines | 192 | 9,13E-04 | ABCE1,ACER3,ACKR3,ACSL4,ADI1,ADM,AHSA1,AIMP1,AKAP12,ATF3,ATM,ATP1B3,ATR,AURKA,BACH1,BCLAF1,BIRC5,BMPR1B,BNIP2,BNIP3,BNIP3L,BPIFA1,BRCA2,CALB1,CASP3,CASP7,CCDC80,CCNB1,CCNG1,CD74,CD82,CDC6,CDCA2,CDH1,CDK1,CDK6,CENPE,CHKA,CKAP2,CLASP1,COL4A1,COL4A2,COPS5,COX6B1,CRYAB,CTNNB1,CYCS,DEPDC1,DHPS,DLL1,DPP4,DUSP4,DYNLL1,ENO1,EPAS1,EPHA2,EPHB2,ERBB4,EREG,FAU,FGFR1,FKBP5,FLNB,GADD45A,GADD45B,GADD45G,GAS1,GLIS3,GLO1,GLS,GRB10,GRK5,HGF,HIF1A,HK2,HNRNPA1,HSPB8,IGF1R,IGF2BP2,IMMT,IRAK2,ITGB1,ITGB3BP,ITGB4,JAG1,JUN,KIF14,KIR3DL1,L1CAM,LDHA,LGALS1,LGALS3,LGALS9,LIMS1,LTV1,MAD2L1,MAF,MAP2K1,MAP3K8,MAPK10,MAPT,MDGA2,MEF2A,MIB1,MIF,MSH2,MSTN,MUC1,MUC16,MYBL1,NDC80,NDRG1,NDUFAF4,NEK2,NOS2,NR4A2,NUAK1,NUF2,PBK,PDHA1,PDK1,PEBP1,PIK3CA,PLD1,PLK4,POLE2,PPIA,PRKAR1A,PRMT1,PSMD4,PTTG1,RALB,RASA1,RBX1,REPS2,RHOJ,RPS19,RPS6KA3,RRM1,RTN1,S100A4,S1PR3,SERPINA3,SFRP4,SHC1,SHQ1,SHROOM2,SLC25A5,SLC9A3R1,SLPI,SMARCA1,SNAI2,SPAG5,SPN,SPOCK1,SPRY2,STK17A,STOML2,STX6,TBK1,TIAM1,TIAM2,TIMP1,TLR3,TLR9,TMBIM4,TMSB10/TMSB4X,TNFRSF10B,TNKS,TOMM20,TOPBP1,TOX,TPT1,TRAF6,TSG101,TTK,TYMS,UACA,UBA2,UCHL5,UGCG,URI1,USP12,USP17L2 (includes others),UTP18,UXT,VHL,WWC1,YAP1,ZBED6,ZEB1,ZFP36 |
| Cell Death and Survival | apoptosis | Apoptosis | 369 | 6,62E-04 | ABCE1,ACER3,ACKR3,ACSL4,ADA,ADAM10,ADI1,ADM,ADRA1A,AHSA1,AIMP1,AKAP12,AMOTL1,ANTXR2,ARG2,ARID5B,ARRB1,ASIC3,ATF3,ATG16L1,ATM,ATP1B3,ATP6AP2,ATR,AURKA,B4GALT5,BACH1,BCLAF1,BEX3,BIRC5,BLVRA,BMPR1A,BMPR1B,BNIP2,BNIP3,BNIP3L,BPIFA1,BRCA2,BTG1,C3AR1,CALB1,CAMK2D,CAPN3,CAPRIN2,CASP3,CASP7,CBFB,CCDC80,CCL3L3,CCL4,CCL5,CCNA2,CCNB1,CCNC,CCNG1,CCR7,CD151,CD164,CD38,CD5L,CD74,CD82,CDC42EP3,CDC6,CDCA2,CDH1,CDH5,CDK1,CDK6,CDKN3,CENPE,CHKA,CIITA,CIRBP,CKAP2,CLASP1,COL4A1,COL4A2,COPS5,COX6B1,CREBL2,CRYAB,CTNNB1,CUL5,CWC15,CYCS,CYP2F1,DACH1,DCK,DECR1,DEPDC1,DHPS,DLL1,DPP4,DRAXIN,DUSP4,DUSP5,DUSP6,DYNLL1,ECT2,EDNRB,EIF2S1,ELMO1,ENO1,EPAS1,EPHA2,EPHB2,ERBB4,ERCC6L,EREG,ERRFI1,ETV5,FAM162A,FAU,FBL,FEN1,FGFR1,FH,FHL1,FIGNL1,FKBP5,FLNB,FOXF2,FUS,G2E3,GADD45A,GADD45B,GADD45G,GAS1,GBE1,GFPT1,GLIS3,GLO1,GLS,GMCL1,GMNN,GNAO1,GNL3,GNPNAT1,GPI,GRB10,GRK5,H1-0,HAPLN1,HEY1,HGF,HIF1A,HK1,HK2,HLA-DMA,HMGA2,HNRNPA1,HOMER1,HSPB8,ID2,IFT57,IFT88,IGF1R,IGF2BP2,IMMT,IRAK2,ISG15,ITGA6,ITGB1,ITGB3BP,ITGB4,JAG1,JUN,KCNH8,KIF14,KIF18A,KIR2DL1/KIR2DL3,KIR3DL1,L1CAM,LAMTOR5,LDHA,LDLR,LGALS1,LGALS3,LGALS9,LGR4,LIMS1,LRP8,LTV1,MAD2L1,MAF,MALT1,MAP2K1,MAP3K20,MAP3K8,MAPK10,MAPT,MDGA2,MDH1,MEF2A,MGP,MIB1,MIF,MIS18A,MNAT1,MSH2,MSH6,MSTN,MUC1,MUC16,MX1,MYBL1,MYDGF,MYSM1,NAE1,NAT8B,NCF1,NCK1,NDC80,NDRG1,NDUFAF4,NEK2,NFYA,NGEF,NLRP2B,NOS2,NR4A2,NR4A3,NUAK1,NUF2,OXR1,PARL,PBK,PCP4,PDHA1,PDK1,PDYN,PEBP1,PELI2,PHLDA1,PIAS2,PIK3CA,PLD1,PLK4,PLP1,POLE2,PON2,PPIA,PPP1CC,PPP2R1B,PPP2R5C,PPP3CA,PPP3R1,PRDX3,PRDX4,PRKAR1A,PRMT1,PSMB1,PSMD4,PSMD6,PTTG1,RAB28,RALB,RAP1B,RASA1,RASSF5,RBM3,RBX1,REPS2,RHOJ,ROCK1,RPS19,RPS3A,RPS6,RPS6KA2,RPS6KA3,RRBP1,RRM1,RRM2B,RTN1,RYBP,S100A14,S100A4,S1PR1,S1PR3,SBNO1,SCG2,SEMA7A,SERPINA3,SERPINI1,SFRP4,SH3KBP1,SHC1,SHC4,SHQ1,SHROOM2,SLC20A1,SLC25A5,SLC4A7,SLC6A6,SLC7A11,SLC9A3R1,SLK,SLPI,SMARCA1,SMARCA5,SNAI2,SPAG5,SPN,SPOCK1,SPRY2,SPRY4,ST18,STK17A,STOML2,STX6,SUMO1,SWAP70,TBK1,TEAD1,TFAM,THAP12,TIAM1,TIAM2,TIGAR,TIMP1,TLR3,TLR9,TMBIM4,TMEM14A,TMF1,TMSB10/TMSB4X,TNFRSF10B,TNKS,TOMM20,TOPBP1,TOX,TOX3,TPT1,TRAF5,TRAF6,TRIB2,TSG101,TTK,TWSG1,TYMS,UACA,UBA2,UBE2M,UBE2V2,UCHL5,UGCG,UNC13B,URI1,USP12,USP17L2 (includes others),USP38,USP47,UTP18,UXT,VAV3,VHL,VTI1B,WDR26,WWC1,XCL1,YAP1,YBX3,YES1,ZBED6,ZEB1,ZFP36,ZFP36L2,ZHX2 |
| Neurological Disease | movement disorder | Movement Disorders | 183 | 4,14E-06 | ABCA5,ACAT1,ACP1,ADCY2,ADRA1A,AFG3L2,ANKH,APLP2,AQP4,ARRB1,ATM,ATP2B1,ATP5MG,ATP6AP2,ATP6V1B2,B3GNT2,B4GALT5,BCAS1,BCHE,CA12,CA13,CA8,CALB1,CAPN3,CASP3,CASP7,CASQ1,CCL4,CCL5,CD38,CD74,CIITA,CIRBP,CNTN1,COX5B,COX7B,COX7C,CRIM1,CRYAB,CTNNB1,CYCS,CYP51A1,DDHD2,DECR1,DGKB,DHCR7,DHPS,DIRAS2,DUSP5,EIF3E,EIF3K,EIF4A2,ELMO1,EPHB2,ERLIN2,ERRFI1,ETV4,ETV5,FAM107A,FAM171A1,FBL,FCGR1B,FDFT1,FRMD4B,FUS,GADD45A,GALC,GAS1,GFAP,GHITM,GLDN,GLS,GNAO1,GNG7,GPI,GPR88,GRK5,GTF3A,HLA-DQA1,HLA-DRA,HOMER1,HPRT1,HTR2A,HTR3E,IER5,IFNGR2,IGSF5,IREB2,ITGB4,JUN,KCNA4,KCNK2,KCNN3,KIF14,KLRC3,L1CAM,LDHA,LDLR,LPL,LRP8,MALT1,MAP3K20,MAPT,MBNL2,MDH1,MEIS1,MGAT2,MT1X,MYBPC1,NAB1,NDRG1,NDUFA7,NDUFB5,NDUFS3,NDUFS4,NDUFS5,NGEF,NHSL2,NOS2,NR4A2,OPN1LW,OXR1,P4HA1,PACRG,PAQR6,PARL,PDK1,PDYN,PEBP1,PGK1,PHKA1,PIAS2,PIP4P2,PLP1,PLSCR4,POLR2I,PPIA,PPM1B,PPP3CA,PPP3R1,PSMB6,PTPRD,PTS,RAB11A,RNF114,RPH3A,RPL17,RPS3A,RPS4X,RPSA,RTN1,S1PR1,SACS,SCG2,SCN4B,SEPTIN6,SERPINA3,SERPINI1,SLC12A2,SLC16A9,SLC6A9,SLIRP,SNCAIP,SOCS5,STARD4,STRN3,SUCLA2,TFAM,TIMP1,TOMM20,TOMM70,TOX3,TPI1,TPT1,TRAF6,TRAM1,TSN,TUBB4B,TWSG1,UCK2,UGCG,USP13,WDR26 |
| Cell Death and Survival | cell death | Cell death of tumor cell lines | 251 | 1,45E-05 | ABCE1,ACER3,ACKR3,ACSL4,ADAM10,ADI1,ADM,AHSA1,AIMP1,AKAP12,ANTXR2,ATF3,ATM,ATP1B3,ATP2B1,ATP5F1A,ATR,AURKA,BACH1,BCHE,BCLAF1,BIRC5,BMPR1B,BNIP2,BNIP3,BNIP3L,BPIFA1,BRCA2,BTG1,CALB1,CAPN3,CASP3,CASP7,CCDC80,CCNB1,CCNG1,CCT8,CD74,CD82,CDC6,CDCA2,CDH1,CDK1,CDK6,CENPE,CHKA,CHSY1,CKAP2,CLASP1,COL4A1,COL4A2,COPS5,COX6B1,CRYAB,CTNNB1,CYCS,DCK,DCTD,DEPDC1,DHCR7,DHPS,DLL1,DPP4,DUSP4,DYNLL1,EIF2S1,ENO1,EPAS1,EPHA2,EPHB2,ERBB4,EREG,FANCL,FANCM,FAU,FDFT1,FEN1,FGFR1,FKBP5,FLNB,FRMD6,GADD45A,GADD45B,GADD45G,GAS1,GBE1,GLIS3,GLO1,GLS,GNAO1,GPI,GRB10,GRK5,HGF,HIF1A,HK1,HK2,HNRNPA1,HSPB11,HSPB8,IGF1R,IGF2BP2,IGFBP2,IMMT,IRAK2,ITGA6,ITGB1,ITGB3BP,ITGB4,JAG1,JUN,KIF14,KIR2DL1/KIR2DL3,KIR3DL1,L1CAM,LDHA,LGALS1,LGALS3,LGALS9,LIMS1,LTV1,MAD2L1,MAF,MAP2K1,MAP3K8,MAPK10,MAPT,MDGA2,MED6,MEF2A,MIB1,MIF,MSH2,MSI2,MSTN,MT1X,MUC1,MUC16,MYBL1,NDC80,NDRG1,NDUFAF4,NEK2,NOS2,NR4A2,NR4A3,NUAK1,NUF2,OAZ1,OXR1,PBK,PDHA1,PDK1,PEBP1,PIK3CA,PLD1,PLEKHA7,PLK4,POLE2,PPAT,PPIA,PPP2R1B,PRKAR1A,PRMT1,PSMD14,PSMD4,PTTG1,RAD51C,RALB,RASA1,RBM43,RBX1,REPS2,RHOJ,RPAP3,RPL27A,RPS19,RPS6KA3,RRM1,RRM2B,RTN1,S100A4,S1PR3,SERPINA3,SERPINI1,SESN2,SFRP4,SH2B3,SHC1,SHQ1,SHROOM2,SLC25A5,SLC7A11,SLC9A3R1,SLPI,SMARCA1,SNAI2,SNCAIP,SNX4,SPAG5,SPN,SPOCK1,SPRY2,STK17A,STOML2,STX6,SUMO1,TBK1,TIAM1,TIAM2,TIMP1,TLR3,TLR9,TMBIM4,TMCC3,TMSB10/TMSB4X,TNFRSF10B,TNKS,TOMM20,TOPBP1,TOX,TOX3,TPT1,TRAF6,TSG101,TTK,TYMS,UACA,UBA2,UBE2C,UBE2K,UCHL5,UGCG,UQCRFS1,URI1,USP12,USP17L2 (includes others),USP47,UTP18,UXT,VAV3,VCAM1,VHL,VRK1,WWC1,YAP1,YEATS4,ZBED6,ZEB1,ZFP36,ZMYND10 |
| Cell Death and Survival | necrosis | Necrosis | 397 | 2,04E-06 | ABCC9,ABCE1,ACAT1,ACER3,ACKR3,ACP1,ACSL4,ADA,ADAM10,ADI1,ADM,ADRA1A,AHSA1,AIMP1,AKAP12,AMOTL1,ANTXR2,ARG2,ARID5B,ARRB1,ATF3,ATG16L1,ATM,ATP1B3,ATP2B1,ATP5F1A,ATP6AP2,ATR,AURKA,B4GALT5,BACH1,BCHE,BCLAF1,BEX3,BIRC5,BMPR1A,BMPR1B,BNIP2,BNIP3,BNIP3L,BPIFA1,BRCA2,BTG1,C3AR1,CACNB2,CALB1,CAMK2D,CAPN3,CASP3,CASP7,CCDC80,CCL3L3,CCL4,CCL5,CCNB1,CCNC,CCNG1,CCR7,CCT8,CD151,CD34,CD38,CD5L,CD74,CD82,CDC42EP3,CDC6,CDCA2,CDH1,CDH5,CDK1,CDK6,CENPE,CHKA,CHMP4B,CHSY1,CIITA,CKAP2,CLASP1,CLEC18B,CLOCK,CNBP,COL4A1,COL4A2,COPS5,COX6B1,CREBL2,CRYAB,CTNNB1,CWC15,CX3CR1,CYCS,CYP2F1,DCK,DCTD,DECR1,DEPDC1,DHCR7,DHPS,DLL1,DPP4,DRAXIN,DUSP4,DUSP5,DUSP6,DYNLL1,EBNA1BP2,EDNRB,EIF2S1,EIF3E,EMP1,ENO1,EPAS1,EPHA2,EPHB1,EPHB2,ERBB4,EREG,FAM162A,FANCL,FANCM,FAU,FDFT1,FEN1,FGFR1,FH,FHL1,FKBP5,FLNB,FOXF2,FRMD6,FUS,GADD45A,GADD45B,GADD45G,GAS1,GBE1,GFAP,GFPT1,GLIS3,GLO1,GLS,GNAO1,GNL3,GNPNAT1,GPI,GPNMB,GPR39,GRB10,GRK5,GUCY1A1,HGF,HIF1A,HK1,HK2,HLA-DMA,HMGA2,HNRNPA1,HPRT1,HSPB11,HSPB8,ID2,IGF1R,IGF2BP2,IGFBP2,IKZF2,IMMT,IRAK2,ISG15,ITGA6,ITGB1,ITGB3BP,ITGB4,JAG1,JUN,KIF14,KIR2DL1/KIR2DL3,KIR3DL1,KIR3DL2,L1CAM,LDHA,LDLR,LGALS1,LGALS3,LGALS9,LIMS1,LTV1,MAD2L1,MAF,MALT1,MAP2K1,MAP3K8,MAPK10,MAPT,MDGA2,MDH1,MED6,MEF2A,MGAT5,MGP,MIB1,MIF,MNAT1,MSH2,MSI2,MSTN,MT1X,MUC1,MUC16,MX1,MYBL1,MYSM1,NAE1,NCF1,NCK1,NDC80,NDRG1,NDUFAB1,NDUFAF4,NDUFS4,NEK2,NFYA,NOS2,NR2C1,NR4A2,NR4A3,NRN1,NUAK1,NUF2,NUP107,NUP54,OAZ1,OXR1,PARL,PBK,PCP4,PDE7B,PDHA1,PDK1,PEBP1,PELI2,PHLDA1,PIK3CA,PLD1,PLEKHA7,PLK4,PLP1,POLE2,PON2,POSTN,PPAT,PPIA,PPP1CC,PPP2R1B,PPP3CA,PPP3R1,PRDX3,PRKAR1A,PRMT1,PSMA3,PSMA4,PSMA7,PSMB1,PSMB3,PSMB5,PSMC5,PSMC6,PSMD14,PSMD4,PSMD6,PTTG1,RAB28,RAD51C,RALB,RASA1,RASSF5,RBM3,RBM39,RBM43,RBX1,RECQL,REPS2,RHOJ,ROCK1,RPAP3,RPL27A,RPL35A,RPL5,RPS15A,RPS16,RPS19,RPS3A,RPS5,RPS6,RPS6KA2,RPS6KA3,RPS7,RPSA,RRM1,RRM2B,RTN1,S100A4,S1PR1,S1PR3,SCG2,SEMA7A,SERPINA3,SERPINI1,SESN2,SFRP4,SH2B3,SH3KBP1,SHC1,SHQ1,SHROOM2,SLC20A1,SLC25A5,SLC39A7,SLC6A6,SLC7A11,SLC9A3R1,SLK,SLPI,SMARCA1,SNAI2,SNCAIP,SNX4,SPAG5,SPN,SPOCK1,SPRR1A,SPRY2,STK17A,STOML2,STX6,STXBP3,SUMO1,SWAP70,TAF1B,TBK1,TFAM,TIAM1,TIAM2,TIGAR,TIMP1,TLR3,TLR9,TMBIM4,TMCC3,TMSB10/TMSB4X,TNFRSF10B,TNKS,TOMM20,TOPBP1,TOX,TOX3,TPT1,TRAF5,TRAF6,TRIB2,TSG101,TTK,TWSG1,TYMS,UACA,UBA2,UBE2C,UBE2K,UBE2M,UBE2V2,UCHL5,UGCG,UNC13B,UQCRFS1,URI1,USP12,USP17L2 (includes others),USP38,USP47,UTP18,UXT,VAV3,VCAM1,VHL,VRK1,VTI1B,WIPF1,WWC1,XCL1,YAP1,YEATS4,YES1,ZBED6,ZEB1,ZFP36,ZHX2,ZMYND10 |
